# Wide-scale identification of novel/eliminated genes responsible for evolutionary transformations

**DOI:** 10.1186/s13062-023-00405-6

**Published:** 2023-08-11

**Authors:** Vassily A. Lyubetsky, Lev I. Rubanov, Maria B. Tereshina, Anastasiya S. Ivanova, Karina R. Araslanova, Leonid A. Uroshlev, Galina I. Goremykina, Jian-Rong Yang, Vladimir G. Kanovei, Oleg A. Zverkov, Alexander D. Shitikov, Daria D. Korotkova, Andrey G. Zaraisky

**Affiliations:** 1 Institute for Information Transmission Problems of the Russian Academy of Sciences (Kharkevich Institute), 19 Build. 1, Bolshoy Karetny per., Moscow, Russia 127051; 2 Department of Mechanics and Mathematics, Lomonosov Moscow State University, Kolmogorova Str., 1, Moscow, Russia 119234; 3 Shemyakin-Ovchinnikov Institute of Bioorganic Chemistry, Russian Academy of Sciences, 16/10, Miklukho-Maklaya Str., Moscow, Russia 117997; 4 Pirogov Russian National Research Medical University, Moscow, Russia; 5 Department of Molecular Medicine, The Scripps Research Institute, La Jolla, USA; 6 Engelhardt Institute of Molecular Biology, Russian Academy of Sciences, 32, Vavilova Str., Moscow, Russia 119991; 7 Plekhanov Russian University of Economics, Stremyanny Lane 36, Moscow, Russia; 8 Advanced Medical Technology Center, The First Affiliated Hospital, Zhongshan School of Medicine, Sun Yat-sen University, Guangzhou, 510080 China; 9 Department of Genetics and Biomedical Informatics, Zhongshan School of Medicine, Sun Yat-sen University, Guangzhou, 510080 China; 10 Present Address: Global Health Institute, School of Life Sciences, EPFL, Lausanne, Switzerland

**Keywords:** Loss or emergence of genes, Loss or emergence of the phenotypic traits, Changes of gene expression, *Xenopus* tadpoles tail regeneration, Gene regulators of regeneration, Gene knockdown, Fast algorithm, Effective computer program

## Abstract

**Background:**

It is generally accepted that most evolutionary transformations at the phenotype level are associated either with rearrangements of genomic regulatory elements, which control the activity of gene networks, or with changes in the amino acid contents of proteins. Recently, evidence has accumulated that significant evolutionary transformations could also be associated with the loss/emergence of whole genes. The targeted identification of such genes is a challenging problem for both bioinformatics and evo-devo research.

**Results:**

To solve this problem we propose the WINEGRET method, named after the first letters of the title. Its main idea is to search for genes that satisfy two requirements: first, the desired genes were lost/emerged at the same evolutionary stage at which the phenotypic trait of interest was lost/emerged, and second, the expression of these genes changes significantly during the development of the trait of interest in the model organism. To verify the first requirement, we do not use existing databases of orthologs, but rely purely on gene homology and local synteny by using some novel quickly computable conditions. Genes satisfying the second requirement are found by deep RNA sequencing. As a proof of principle, we used our method to find genes absent in extant amniotes (reptiles, birds, mammals) but present in anamniotes (fish and amphibians), in which these genes are involved in the regeneration of large body appendages. As a result, 57 genes were identified. For three of them, *c-c motif chemokine 4*, *eotaxin-like*, and a previously unknown gene called here *sod4*, essential roles for tail regeneration were demonstrated. Noteworthy, we established that the latter gene belongs to a novel family of Cu/Zn-superoxide dismutases lost by amniotes, SOD4.

**Conclusions:**

We present a method for targeted identification of genes whose loss/emergence in evolution could be associated with the loss/emergence of a phenotypic trait of interest. In a proof-of-principle study, we identified genes absent in amniotes that participate in body appendage regeneration in anamniotes. Our method provides a wide range of opportunities for studying the relationship between the loss/emergence of phenotypic traits and the loss/emergence of specific genes in evolution.

**Supplementary Information:**

The online version contains supplementary material available at 10.1186/s13062-023-00405-6.

## Background

Changes in genotypes underlying the major evolutionary transformations associated with the emergence of extant taxa of vertebrates remain poorly understood. In particular, such transformations include the emergence in amphibian ancestors of limbs adapted for terrestrial walking and the appearance in birds and mammals of the ability to maintain a constant body temperature, the progressive development of the dorsal part of the telencephalon in birds and mammals, and a decrease in the ability to regenerate limbs compared to that of fish and amphibians.

It is generally accepted that most evolutionary transformations at the phenotype level are associated with rearrangements of genomic regulatory elements that control the operation of gene networks and consist of approximately the same set of genes in representatives of different classes of animals [26, 39, 53]. Meanwhile, in rarer cases, significant evolutionary transformations could also be associated with the loss or emergence of genes. [8, 18, 45, 47, 54].

For example, the emergence of a new homeobox gene, *anf/hesx1*, in the ancestors of extant vertebrates, probably due to the fusion of parts of two homeobox genes of different classes, led to prerequisites for the development of a unique brain unit, the telencephalon, in vertebrates [2, 13, 28, 58]). The loss of *actinodins* by tetrapod ancestors could have contributed to the development of limbs adapted for terrestrial walking [59]. The loss of *c-answer*, *ag1*, *ras-dva1*, and *tfp4* by the ancestors of warm-blooded animals could be one of the reasons for the loss of their strong ability to regenerate limbs, which is typical of many extant cold-blooded animals that have retained these genes [20–23, 30, 57]. One may assume in this regard that a targeted search and study of genes that were lost or emerged at evolutionary stages accompanied by major phenotypic transformations could be an effective approach to understanding the genetic causes of such transformations.

Previously, we created a method that includes an algorithm and a computer program that allows one to search for genes that were lost or emerged at any given stage of evolution [30, 51]. In the present work, we have developed a new targeted search method for genes such that their loss/emergence at a given stage of evolution could underlie the loss/acquisition of certain traits in large taxa that emerged in the later evolution of this stage. This method represents a combination of two components: (i) a large-scale search for genes that were lost/emerged at the evolutionary stage when the trait of interest was lost/emerged, and (ii) a search by deep RNA sequencing for genes that are differentially expressed during the development of this trait of interest in a model organism. Another important innovation used in this method is an original algorithm for a large-scale search for orthologous genes in several evolutionarily distant organisms using criteria of homology and local genomic synteny. We called this method WINEGRET, first, after the first letters of the words in its brief description, wide-scale identification of novel or eliminated genes responsible for evolutionary transformations; and second, because the similarity of this name to the word vinaigrette emphasizes the fact that our method is a combination of two main components: a screening for the lost/emerged genes and a search for the differentially expressed genes.

As a proof of principle, we used this method to identify genes that simultaneously satisfy two conditions: (1) such genes should be lost by reptiles, birds, and mammals but preserved in fish and amphibians; and (2) they should significantly change their expression levels during regeneration of the body appendages in a model species chosen from among fish and amphibians. As an example, the frog *Xenopus tropicalis* was chosen, the tadpoles of which demonstrate a high capability to regenerate amputated tail tips. Ensuring these two conditions resulted in a short list of 57 genes, 30 of which are significantly upregulated during regeneration, while 27 are downregulated. We demonstrated that the activity of at least three of the upregulated genes, *c-c motif chemokine 4*, *eotaxin-like*, and a previously unknown gene called here *sod4* are essential for tail regeneration. Noteworthy, we established that the latter gene belongs to a novel family of Cu/Zn-superoxide dismutases lost by amniotes, SOD4.

In sum, the WINEGRET method provides wide opportunities for studying relationships between the loss/emergence of specific genes and the loss/emergence of phenotypic traits in evolution.

## Results

### Description of the WINEGRET method

The proposed WINEGRET method consists of four tasks (Task I, Task II, Task III and Task IV), and when it is applied to any two sets of species that are contrasted to each other according to a certain trait of interest, it allows one to identify genes that were lost/emerged during evolution in only one of these sets of species, and at the same time participate in manifesting the trait in species that have it. In particular, as a proof of principle, we identified 57 genes absent in reptiles, birds, and mammals but present in fish and amphibians whose expression changed during tadpole tail regeneration in a model representative of amphibians, the frog *Xenopus tropicalis*.

#### Task I

The goal of Task I is to find genes whose loss/emergence in evolution correlates with the loss/emergence of the trait of interest. To this end, two sets of species should be chosen in advance: (1) species in which this trait is definitely present and (2) species in which it is definitely absent. In many cases, the trait of interest was lost/emerged at a certain stage of evolution during the transition between two sets of large taxa that emerged successively in evolution. In such cases, species from the taxa that emerged earlier can be designated *lower*, and species from the taxa that emerged later can be designated *upper*. The species that arose between the lower and upper ones are designated *middle*.

**Fig. 1:**
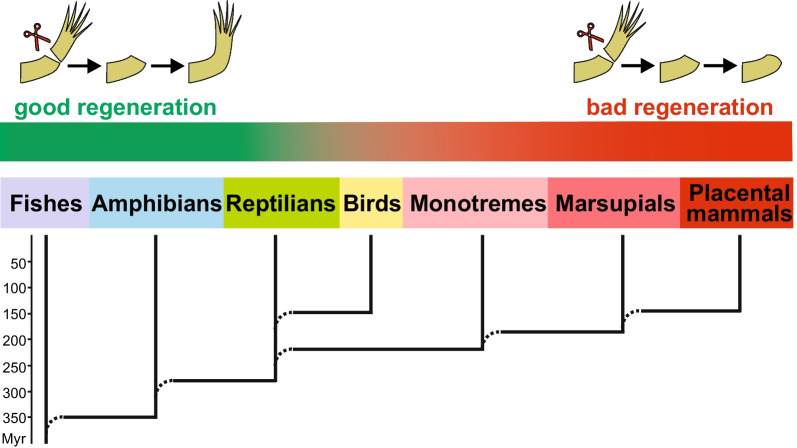
Declining capability to regenerate large body appendages, i.e., limbs and tail, in the line of vertebrates from fish and amphibians to placental mammals. Shown are groups of vertebrates that emerged sequentially in evolution. Some extant species from these groups were collected into the lower, middle, and upper species sets to search for genes absent in reptiles, birds, and mammals but involved in tadpole tail regeneration in *Xenopus tropicalis*, which was chosen as the reference species *R* (see main text for details)

In our proof-of-principle study, the trait of interest is the *regeneration of large body appendages*; this ability is strong in fish and amphibians but completely lost in placental mammals [29, 38] (Fig. 1).

As a specific model of such regeneration, we chose the process of restoring the tip of the *Xenopus tropicalis* tadpole tail after its amputation. Accordingly, in our case, the *lower* species are those belonging to the fish and amphibian groups, and the *upper* ones are species belonging to the placental mammal group (see the list of selected species in Table 1).

**Table 1:** The complete genomes of 33 species (lower, middle and upper) used in this study

Scientific name	Common name	Assembly	Accession
Fish (lower spp.)			
*Callorhinchus milii*	Elephant shark	IMCB_Cmil_1.0	GCF_018977255.1
*Danio rerio*	Zebrafish	GRCz11	GCF_000002035.6
*Latimeria chalumnae*	Coelacanth	LatCha1	GCF_000225785.1
*Oreochromis niloticus*	Nile tilapia	O_niloticus_UMD_NMBU	GCF_001858045.2
*Oryzias latipes*	Japanese medaka	ASM223467v1	GCF_002234675.1
*Takifugu rubripes*	Fugu	fTakRub1.2	GCF_901000725.2
Amphibians (lower spp.)			
*Xenopus tropicalis*			
(Reference species)	Tropical clawed frog	UCB_Xtro_10.0	GCF_000004195.4
Reptilians (middle spp.)			
*Anolis carolinensis*	Anole lizard	AnoCar2.0	GCF_000090745.1
*Chrysemys picta bellii*	Painted turtle	Chrysemys_picta_bellii-3.0.4	GCF_000241765.4
*Crocodylus porosus*	Australian saltwater crocodile	CroPor_comp1	GCF_001723895.1
*Notechis scutatus*	Mainland tiger snake	TS10Xv2-PRI	GCF_900518725.1
*Pelodiscus sinensis*	Chinese softshell turtle	PelSin_1.0	GCF_000230535.1
*Pogona vitticeps*	Central bearded dragon	pvi1.1	GCF_900067755.1
Birds (middle spp.)			
*Anas platyrhynchos*	Mallard	ZJU1.0	GCF_015476345.1
*Gallus gallus*	Chicken	bGalGal1.pat.whiteleghornlayer.GRCg7w	GCF_016700215.2
*Meleagris gallopavo*	Turkey	Turkey_5.1	GCF_000146605.3
*Taeniopygia guttata*	Zebra Finch	bTaeGut1_v1.4.pri	GCF_003957565.2
Mammals			
Monotremata (middle spp.)			
*Ornithorhynchus anatinus*	Platypus	mOrnAna1.pri.v4	GCF_004115215.2
Marsupialia (middle spp.)			
*Monodelphis domestica*	Gray short-tailed opossum	MonDom5	GCF_000002295.2
*Sarcophilus harrisii*	Tasmanian devil	mSarHar1.11	GCF_902635505.1
Placentalia (upper spp.)			
*Bos taurus*	Cow	ARS-UCD1.3	GCF_002263795.2
*Canis lupus familiaris*	Dog	Dog10K_Boxer_Tasha	GCF_000002285.5
*Equus caballus*	Horse	EquCab3.0	GCF_002863925.1
*Felis catus*	Cat	F.catus_Fca126_mat1.0	GCF_018350175.1
*Gorilla gorilla*	Gorilla	Kamilah_GGO_v0	GCF_008122165.1
*Heterocephalus glaber*	Naked mole-rat	HetGla_female_1.0	GCF_000247695.1
*Homo sapiens*	Human	GRCh38.p14	GCF_000001405.40
*Loxodonta africana*	African bush elephant	Loxafr3.0	GCF_000001905.1
*Macaca mulatta*	Macaque	Mmul_10	GCF_003339765.1
*Mesocricetus auratus*	Golden Hamster	BCM_Maur_2.0	GCF_017639785.1
*Mus musculus*	Mouse	GRCm39	GCF_000001635.27
*Pan troglodytes*	Chimpanzee	Clint_PTRv2	GCF_002880755.1
*Sus scrofa*	Pig	Sscrofa11.1	GCF_000003025.6

Additionally, among the selected species, it is necessary to choose one species with a well-annotated genome, further called the *reference* species and *denoted R*. It is convenient if this species is the same as that in which we search for genes whose expression changes in the developmental manifestation of the trait or process of interest, such as the regeneration process in our case. The sought-for genes of *R* should be lost among the species of group (2). When searching for genes that *emerged* in evolution during the transition from the lower species to the upper ones, the species *R* should be selected from among the upper species. If the goal is to search for genes lost by the upper species, the reference species is selected from among the lower species. Since our proof-of-principle study considers only the second case, we chose the frog *Xenopus tropicalis* as the reference species *R*.

Between the lower and upper species, there could be species in which the trait or process of interest is still preserved in some reduced form. We designate these species as *middle* ones. These species may be of interest from the point of view of studying the mechanisms of loss/emergence of a trait or process of interest. This is because the genomes of these species may still contain some genes from the set of genes that distinguish the lower species from the upper species. In our case, the middle species belong to the reptile and bird groups as well as to the infraclasses of monotremes (Monotremata) and marsupials (Marsupialia) among mammals (Fig. 1). Species from these two infraclasses are conveniently grouped as primitive mammals. Of particular interest for our proof-of-principle study are the genes that were lost in all these middle species, as well as in the upper species, but retained in the lower species that have a strong ability to regenerate large body appendages.

To select genes that were preserved during evolution in the species of set (1) and were lost in the species of set (2), it is necessary to perform a search in each species *A* of both sets for orthologs of all protein-coding genes in the reference species *R*. As a result, the genes in *A* for which such orthologs are not found could be considered *lost* in species *A*.

**Fig. 2:**
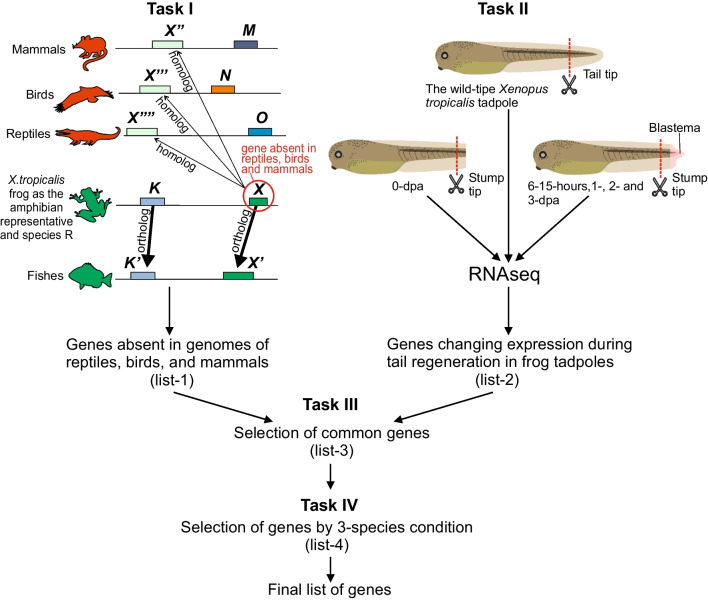
Scheme of the WINEGRET method using the example of a search for genes absent in mammals, birds, and reptiles but present in fish and amphibians and involved in the tail regeneration of *Xenopus tropicalis* frog tadpoles

As opposed to our previous works [30, 51], our new method does not use *candidate orthologs from an online database* when searching for an ortholog of gene *X* from reference species *R* in species *A* (see the first subsection of Discussion below for more details). Instead, a candidate ortholog of gene *X* in species *A* is found by selecting a few homologs *X*′ in *A* that are most similar to *X* in *R* and then testing each *X*′ for local synteny with *X*. If local synteny of *X* is not confirmed for any *X*′, then gene *X* from *R* is postulated to have no orthologs in *A* (see Fig. 2, Task I).

The Task I panel shows a selection of candidates to compile a list-1 (Additional file 1: Table S1) consisting here of genes absent in mammals, birds, and reptiles but present in fish and amphibians. The frog gene *X* is shown, which has an *X*′ ortholog in fish, which is confirmed by the local synteny of these genes, i.e. the presence in their vicinity of mutually orthologous witnesses: genes *K* and *K*′ for *X* and *X*′, respectively. At the same time, *X*, although it has various homologs *X″*, *X‴* and *X⁗* in mammals, birds, and reptiles, does not have local synteny with them: genes *M*, *N*, and *O* from the vicinity of *X″*, *X‴*, and *X⁗* do not have orthologs in the vicinity of *X*. See Results, as well as Methods sections for more detailed conditions on *X*′, *X″*,… In the Task I panel, orthologs are connected by thick arrows, and homologs (non-orthologs) by thin arrows.

The Task II panel shows the scheme of the experiment, the result of which is the list-2 of genes (Additional file 2: Table S2) whose expression is changing significantly at different stages of the tadpole tail regeneration.

The Task III panel shows that the list-3 of genes (Additional file 3: Table S3) is found by selecting genes common to the list-1 and list-2.

The Task IV panel shows the testing by the 3-species condition if there are no orthologs in reptiles, birds, and mammals of fish genes orthologous to genes in list-4 (Additional file 4: Table S4). It results in the final list of genes (Additional file 5: Table S5). See below for details.

**Fig. 3:**
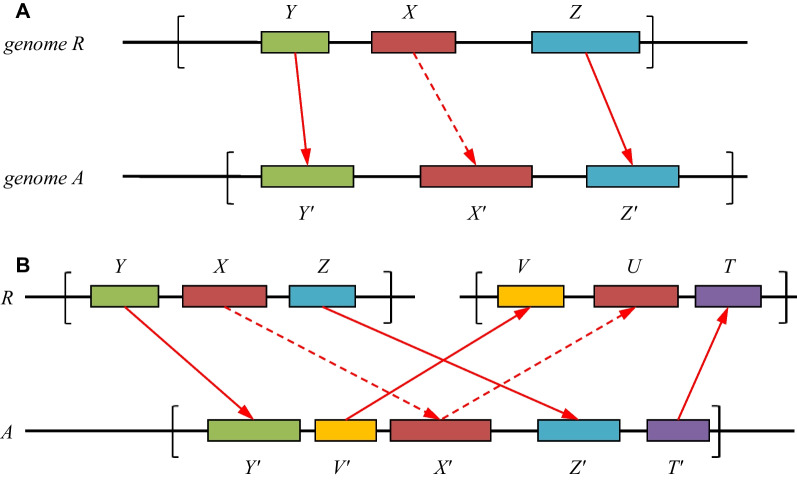
The principles of orthologs search in Task I. **A** Forward check of a homolog *X*′ in species *A* of a given gene *X* in species *R* that involves, e.g., two witnesses: *Y* and *Z* in *R* and *Y*′ and *Z*′ in *A*. The dashed arrow represents the α-homology condition, and the solid arrow represents the β-homology (see for the explanation of α- and β-homology Step 2 in section A of Methods). The brackets indicate the chosen neighborhoods of the genes *X* and *X*′, in which the witnesses are sought. The witnesses may be arbitrarily positioned and directed within the neighborhood. **B** Backward check for the existence of an alternative *U* that is more similar to candidate *X*′ than *X* that involves, e.g., two witnesses. The dashed arrow represents the α-homology condition, and the solid arrow represents the β-homology. The brackets indicate the chosen neighborhoods of the genes *X* and *X*′, in which the witnesses are sought. The witnesses may be arbitrarily positioned and directed within the neighborhood

If the synteny of *X* with one of *X*′ is confirmed (see Fig. 3A), then a *backward check* is performed for *X*′ (see Fig. 3B), during which it is verified whether there is any gene *U* in *R* that is a closer homolog of *X*′ than *X* and has local synteny with *X*′. If such a *U* is found, then it is assumed that *X*′ is actually not an ortholog of *X*. Then, it is also postulated that *X* has no orthologs in *A*. If such a *U* is not found, then *X*′ in *A* is assumed to be an *ortholog* of *X* in *R*.

The selection of the closest homologs *X*′ in species *A* for *X* in the reference species *R* is performed by comparing the protein sequences encoded by *X* with the protein sequences encoded by all genes from *A*. To this end, all homologs of gene *X* are selected in *A* at a deliberately low cut-off, for example, the one that BLAST [4] uses by default. The list of homologs is ordered by decreasing similarity to *X*, and then the first *u* homologs, where *u* is the algorithm parameter, are used in further analysis.

The reason for the development of this method was that the NCBI, Ensembl or similar ortholog in species *A* of gene *X* from the reference species *R* is not always the closest of its homologs [7]. Moreover, the closest homolog is often far from unambiguous; this is what led us to introduce a parameter *u* with a value strictly greater than one. In other words, the selection of homologs at a static cut-off using a single characteristic, e.g., *E*-value or percent identity, does not always adequately predict gene loss. The opportunity to choose an individual value of the parameter *u* > 1 for each species *A* and fixed species *R* is equivalent to using a dynamic cut-off threshold for homologs.

See a more rigorous description of the Task I algorithm, which three steps in Method section.

#### Task II

The goal of this task is to find genes in a selected model organism with a trait of interest whose expression changes significantly at different stages of the developmental manifestation of this trait. For example, we can find genes that change their levels of expression at different stages of the development of some organ or, as in our case, at different stages of the regeneration of an organ after its amputation. It is postulated that a significant change in the level of gene expression indicates the important role of the gene in the development of the trait of interest. The data for searching for such genes can be obtained both in the course of an original experiment comparing tissue transcriptomes taken at different stages of trait development in a selected model organism and as a result of reprocessing raw data previously deposited in open databases.

In our proof-of-principle study, we searched for genes that significantly change expression at different stages of tail regeneration in the *Xenopus tropicalis* frog tadpole. For this, raw data deposited by the authors of [6] in SRP091865 (related to GEO: GSE88975) were analyzed. These are read libraries for the freshly amputated tail tip (WT data) and the tail stump fragments at 0, 6, 15, 24, and 72 h after amputation; see Fig. 2, Task II. To map the reads to the corresponding genes, we aligned these libraries to the UCB_Xtro_10.0 assembly of the *Xenopus tropicalis* genome. The WT data are attributed to a conditional previous point in time. For the obtained data, the analysis of differential gene expression was performed independently using two statistical models: those of [12, 50]. Genes that were found to have a significant change in expression level in at least one pair of time points in the “exact test” model were then double checked for significance against the general linear model and included in list*-*2. This list contains differentially expressed (DE) genes along with an assessment of the significance of their differential expression—the false discovery rate (FDR). List-2 depends on the FDR threshold. Therefore, if FDR < 0.1, then list-2, for our data, contains 3839 genes. If the cut-off is 0.01, then list-2 contains 2007 genes, as shown in Additional file 2: Table S2. The example used the condition FDR < 0.01.

In this task, one can also take into account how much the expression changes; i.e., one can impose a lower limit on the value $\theta = \left| {\log {\text{FC}}} \right|$, where logFC is the binary logarithm of the change in the expression level between two time points (log-fold-change)and $\left| {\, \cdot \,} \right|$ designates the modulus of a real number. However, this is not always necessary. The reason is that for data such as those in our proof-of-principle study (Additional file 2: Table S2), the full list of DE genes with FDR < 0.01 contains 2007 genes with the smallest $\theta$ value of 1.1; i.e., in all these genes, the change in expression was more than twofold. Tasks I and II are independent of each other and can be carried out simultaneously.

#### Task III

The goal of the third task is to obtain list-3, consisting of genes common to list-1 and list-2. Accordingly, in our proof-of-principle study, we first identified a set of frog genes whose expression significantly changes during regeneration of the amputated tail and which are present in fish and amphibians but were lost in placental mammals. These 268 genes, which belong to both list-1 and list-2, are listed in Additional file 3: Table S3 in descending order of logFC to enable the user to select genes within the desired range of $\theta$. For instance, the first 132 genes in Additional file 3: Table S3 are those whose expressions increase more than fourfold, the next 20 genes increase their expression more than twofold, and all subsequent genes decrease their expression by two times or more. Similar to Additional file 1: Table S1, Additional file 3: Table S3 allows the selection of genes lost at any of the evolutionary steps shown in Fig. 1. Thus, by choosing the condition $m = n = p = 0$, we obtained list-4 of 109 genes that are involved in tail regeneration in the frog tadpole and absent in extant reptiles, birds, and mammals (Additional file 4: Table S4). This list is a starting point for Task IV.

#### Task IV

**Fig. 4:**
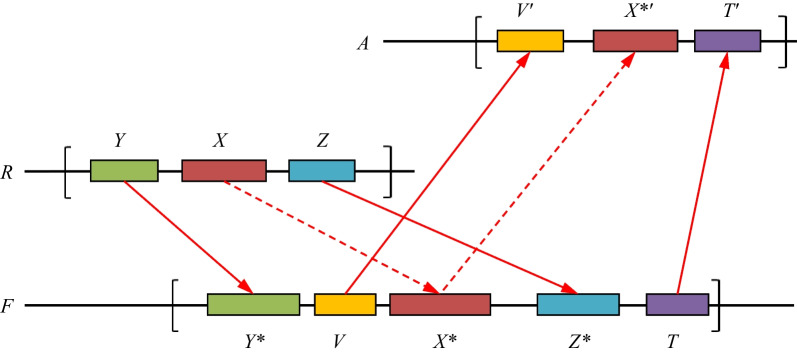
Checking whether gene *X* from *R* has an ortholog X*′ in upper species *A* through a fish genome *F* that involves, e.g., two witnesses. Here, *R* is a fixed reference species; gene *X* has the ortholog X* in fish *F* confirmed by two witness pairs, *Y*–Y* and Z–Z*. The gene X* in turn has the ortholog X*′ in upper species *A* confirmed by two witness pairs, *V–V*′ and *T–T*′. The dashed arrow represents the α-homology condition, and the solid arrow represents the β-homology (see for the determination of α- and β-homology Step 2 in section A of Methods). The brackets indicate the chosen neighborhoods with radii *r*, in which the witnesses are sought. The witnesses may be arbitrarily positioned and directed within the neighborhood

This final task aims to exclude from list-3 of *Xenopus tropicalis* genes those whose orthologs in fishes have orthologs in upper species. The reason for this is that even if gene *X* from Additional file 4: Table S4 has no orthologs in upper species but has ortholog X* in one or more fish species, orthologs in upper species could yet be found for gene X* (Fig. 4). In this case, one cannot consider gene *X* to be eliminated in the upper species compared with the lower species. To exclude such genes from Additional file 4: Table S4, we used the following procedure, which we called *the three-species condition*.

For each amphibian and fish species (other than the reference species *R*) listed in Table 1, we search for ortholog X* for each gene *X* from *R*. This means that conditions (*a*), (*b*), and (*c*) are valid for *X* and X*. Then, we check whether an ortholog X*′ exists in any upper species *A* for X*. This means that the same conditions (*a*), (b), and (*c*) are valid with several given neighborhood radii $r_{1} ,r_{2} , \ldots$. If it appears that an upper species contains the ortholog X*′, then the three-species condition is broken for a given radius of neighborhood. If the condition is broken for all radius values, we exclude *X* from list-4. The result of this procedure we call the *final list*.

Notably, it is possible to verify the three-species condition in Task I in addition to the conditions (*a*), (*b*), and (*c*), but checking it in the separate Task IV is faster and allows one to analyze the excluded genes in more detail.

In our proof-of-principle study, we tested the three-species condition for the following values of neighborhood radii: $r_{1} = 1{\text{ Mbp}}$, $r_{2} = 2{\text{ Mbp}}$, and $r_{3} = 5{\text{ Mbp}}$. If the three-species condition holds for the given gene from Additional file 4: Table S4, then the value 1 appears in Column R, S, or T, depending on the neighborhood radius. Otherwise, the value is zero. We excluded the genes that contained zeros in all these columns. The remaining 57 genes formed the final list of our data (Additional file 5: Table S5). Among these genes, 30 increased their expression during regeneration by more than two times, while 27 decreased their expression. Since elimination during evolution of the latter 27 genes, which are already downregulated in regeneration in anamniotes, was unlikely critical for the loss of regeneration ability in amniotes, one may focus further attention only on the first 30 genes, whose expression is activated during regeneration.

### Testing the roles in tadpole tail regeneration of 3 of the identified 30 genes absent in extant reptiles, birds, and mammals

**Fig. 5:**
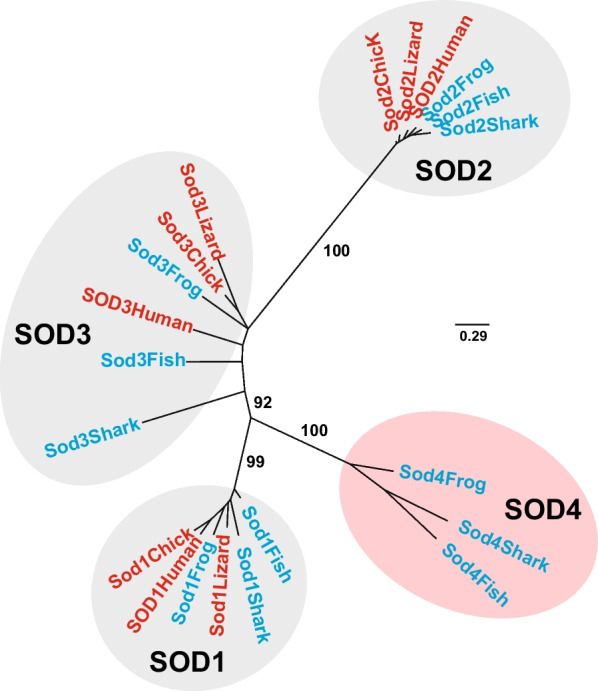
Unrooted phylogenetic tree of SOD1, SOD2, SOD3, and SOD4 family proteins in the following jawed vertebrates: shark (*Callorhinchus milii*), bony fish (*Danio rerio*), frog (*Xenopus tropicalis*), lizard (*Gekko japonicus*), chicken (Gallus gallus) and human (*Homo sapiens*). Protein alignment and phylogenetic tree were built using MAFFT v7.511 [27] and IQ-TREE v2.2.0 [25, 44] tools for proteins under the following accessions: AFM87136.1 for Sod1Shark, NP_571369.1 for Sod1Fish, NP_001016252.1 for Sod1Frog, XP_015271096.1 for Sod1Lizard, NP_990395.2 for Sod1Chick, CAG46542.1 for SOD1Human, NP_001279581.1 for Sod2Shark, NP_956270.1 for Sod2Fish, NP_001005694.1 for Sod2Frog, XP_015268482.1 Sod2Lizard, NP_989542.2 for Sod2Chick, NP_001019636.1 for SOD2Human, AFM90279.1 for Sod3Shark, XP_001332758.1 for Sod3Fish, NP_001106630.1 for Sod3Frog, XP_015272245.1 for Sod3Lizard, XP_040525137.1 for Sod3Chick, CAG46651.1 for SOD3Human, XP_042193029.1 for Sod4Shark, XP_001343650.5 for Sod4Fish, and XP_017953150.2 for Sod4Frog. The branch support values were calculated using the ultrafast bootstrap approximation [43] with 1000 replicates

Since our screening by WINEGRET was based on finding genes lost in amniotes among the list of genes whose expression significantly changes during tadpole tail regeneration (see Additional file 2: Table S2), one may be sure in advance that all genes selected by our program play some role in regeneration. However, to demonstrate this directly, we tested the expression and roles in tail regeneration of the following three genes selected from the identified 30 genes upregulated during regeneration: *c-c motif chemokine 4* (LOC100493779), *eotaxin-like* (LOC101733569), and an unknown protein with three putative superoxide dismutase Cu/Zn-binding domains (LOC100495179). We chose these genes because the first two encode signaling factors of the cytokine family, which are known to play important roles in regeneration. The third gene encodes a protein belonging to an unknown type of animal Cu/Zn superoxide dismutase (Cu/Zn SOD), an enzyme that accelerates the reaction of the superoxide anion (O_2_^•−^) with itself to form hydrogen peroxide (H_2_O_2_), which also plays an important role in regeneration [15, 16, 40]. To clarify the phylogenetic relationships of this *Xenopus tropicalis* LOC100495179 protein with other animal SODs, we aligned it and its orthologs in the shark (*Callorhinchus milii*) and the bony fish (*Danio rerio*) with Cu/Zn-superoxide dismutases of all three known families, SOD1, SOD2, and SOD3, from the same three species and those from the lizard (*Gekko japonicus*), chicken (Gallus gallus) and human (*Homo sapiens*). As a result, we established that the LOC100495179 protein and its orthologs indeed form a novel family of Cu/Zn-superoxide dismutases lost by amniotes. Accordingly, we named this family SOD4 (Fig. 5).

Moreover, our further phylogenetic analysis of proteins revealed that members of the SOD4 family are present in jawless vertebrates (lampreys), in members of different types of Metazoa, in their nearest single-celled relatives Choanoflagellates, and in a sister group of Metazoa and Choanoflagellates, Filasterea, but not in Fungi (Additional file 6: Fig. S1; see also Additional file 7: Fig. S2 for rectangular trees with detailed branch support values).

The common features of SOD4 family proteins are multiple Cu/Zn-superoxide dismutase domains (three in all analyzed vertebrates), a signal peptide on the N-terminus, and a transmembrane domain on the C-terminus. These features indicate that SOD4 proteins operate extracellularly, similar to secreted members of the SOD3 family. However, the C-terminus transmembrane domain indicates that SOD4 proteins are anchored in the cell membrane.

**Fig. 6:**
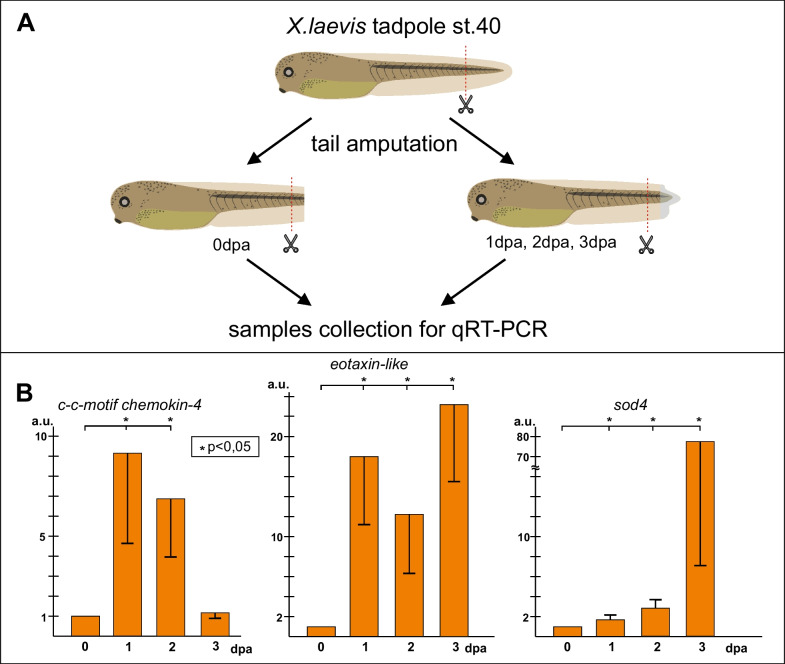
Testing selected gene expression in *Xenopus laevis* tadpole tail regeneration. **A** Schema of experiments. The level of tail amputation is shown by red dotted line. **B** Results of qRT-PCR analysis of the expression of three genes selected from the final list in cells of tip of the amputated tail stump

Since all experiments were performed on *Xenopus laevis* tadpoles, we used the following cDNA sequences of the *Xenopus laevis* orthologs of the three selected *Xenopus tropicalis* genes to design the corresponding primers: *c-c motif chemokine 4* (GenBank: OCT81172.1), *eotaxin-like* (LOC108717158), and *sod4* (LOC121398191). As a result, we confirmed that the expression of all these genes is indeed significantly increased during regeneration while various temporal patterns are demonstrated (Fig. 6).

**Fig. 7:**
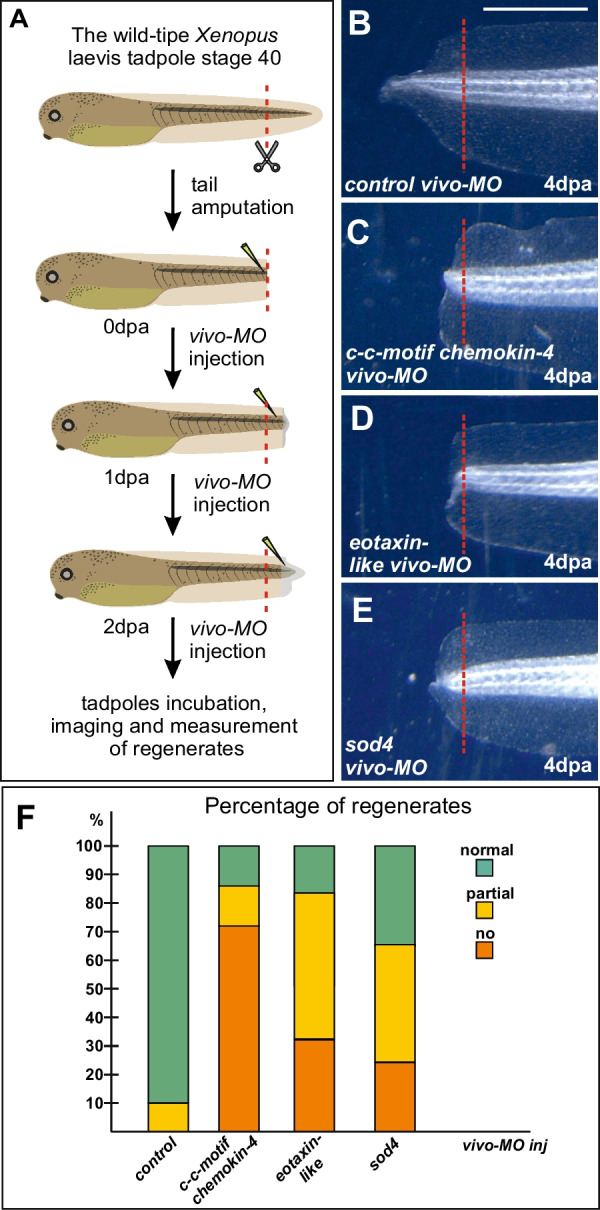
The effect of vivo-MO-induced knockdown of *c-c motif chemokine 4*, *eotaxin-like*, and *sod4* on the regeneration of the amputated *Xenopus laevis* tadpole tail. **A** Schema of experiments. Tail stumps were successively injected at 0, 1 and 2 dpa with either control vivo-MO or with vivo-MO to mRNA of each of the aforementioned three genes. The level of amputation is shown by red line. **B**–**E** Typical samples of tails of *Xenopus laevis* tadpoles injected with the indicated vivo-MO at 4th day after amputation. The level of amputation is shown by red line. **F** Percent distribution of three types of the regenerating tails of *Xenopus laevis* tadpoles (normal regeneration, partial and no regeneration) among the 4 dpa tails injected with the control vivo-MO and vivo-MOs to *c-c motif chemokine 4*, *eotaxin-like*, and *sod4* mRNA. Common scale bar for B–E of 300 microns is shown in B

Then, for three selected genes, *c-c motif chemokine 4*, eotaxin-like, and *sod4*, we tested the effects of their downregulation on tail regeneration by injecting vivo-antisense morpholino (vivo-MO) into their mRNA in the tail stump. In our previous works, we confirmed the effectiveness of vivo-MO in similar experiments [22, 23]. As a result, we observed severe suppression of tail regeneration in all three cases (Fig. 7). Thus, these results demonstrate the essential role of all three tested genes in tail regeneration.

## Discussion

### An effective method for targeted identification of genes that were lost/emerged at a particular evolutionary stage and are associated with a phenotypic trait that was lost/emerged at the same stage

In this work, we present WINEGRET, a method for targeted identification of genes whose loss/emergence in evolution could be associated with the loss/emergence of a phenotypic trait of interest. We developed this method by combining wide-scale genomic bioinformatic screening for genes that were lost/emerged at a given evolutionary stage with screening for genes differentially expressed at different stages during the development of the trait of interest in a model organism having this trait.

The large-scale computer screening of genes that are present in one set of species, which have some morphological or functional trait of interest, and were lost in another set of species, which do not have this trait, is a challenging bioinformatics task. It is assumed that performing this task will yield genes the loss/emergence of which at a given evolutionary stage could provide a basis for the loss/emergence of a trait of interest. To the best of our knowledge, the first works describing a method for performing such a task were [30, 51]. However, the described method had two serious limitations, which our new method does not have.

First, in that method, we tested the preservation of local genomic synteny by relying on potential orthologs that were previously identified in online databases such as Ensembl and GenBank [42, 46] as a result of graph and tree methods. Thus, we initially limited the number of possible genes tested for presence/absence in a genome to the set of potential orthologs contained in these online databases. Note that the problem of orthology inference is quite complicated and still far from being completely solved. Different methods do not always yield consistent orthology predictions, even for evolutionarily close mammals such as mice and rats, and this problem only worsens with increasing evolutionary distance between species. The approaches to solving this problem were reviewed, e.g., in [1, 32, 56]. The results obtained by popular algorithms for orthology inference were compared in [46]. An extensive list of existing orthology databases is supported in the framework of the Quest for Orthologs initiative (https://questfororthologs.org) [37], and very recent advances in the field should also be mentioned [17, 33].

In contrast, in our WINEGRET method, the information on gene orthology is not taken from available databases but generated de novo. In this method, an original algorithm is used that simultaneously includes a search for homologs, verification of synteny, and a backward check of the identified orthologs as well as verification of significant gene expression changes at different stages of the developmental manifestation of a given trait or process. Thus, our novel method can reveal the maximal set of orthologs in large-scale computer screening across many selected genomes by using quickly computable conditions.

Second, our old method described in [30, 51] does not allow one to target the lost/emerged genes associated with a trait of interest but only the genes that were lost/appeared at a given evolutionary stage. In contrast, WINEGRET lacks this flaw because we combine the developed method of searching for genes that were lost/emerged at the evolutionary stage of interest with information about genes differentially expressed during the development of the trait of interest. This combination, for the first time, allows one to find genes that are associated with the trait of interest among those that were lost/emerged at a given evolutionary stage.

In a sum, it is important to note that the WINEGRET method can be used for a very wide range of tasks in which genes involved in the development of various traits that were lost/emerged at a certain evolutionary stage are needed.

### Potential limitations of the WINIGRET method

One limitation of our method is its inability to identify interspecies orthologs lacking local synteny. We estimated this underprediction to be approximately 1.1% on average (see subsection Step3 in section A of Methods and Additional file 8: Appendix 1). Notably, this estimate closely matches the percentage of orthologs lacking local synteny in mammals, which is reported as 1.3% [24].

Fortunately, the impact of this underprediction is minimized within the WINIGRET method, as its primary objective is to define the presence of orthologs of the reference species in two groups of species, considered as a whole. For instance, in our proof-of-principle study, to consider frog’s gene *X* is absent in amniotes, we stipulate that it must be present in at least one fish species but not in any of the considered amniotic species. With this criterion in mind, the probability of none of the orthologs of gene *X* in amniotes (if they indeed exist in amniotes) exhibiting synteny is exceedingly low. This probability can be estimated as *P*^*N*^, where *P* denotes the probability of no synteny between orthologs of the two species, approximately 0.011 or 0.013 (as mentioned above), and *N* is the number of considered amniote species (in our case, 26).

Another constraint of WINIGRET method arises from its inability to recognize multiple orthology when close paralogs originate from the duplication of an ancestral gene and share common witnesses. For instance, considering two such paralogs, *X* and *U*, in the species *R*, our method will select only one (the most homologous to gene *X*′ from the species *A*) as a gene whose ortholog is present in species *A*. At the same time, the second paralog will be deemed absent in *A*. Obviously, this may seem incorrect from the perspective of the widely accepted concept of semantically standardized gene orthology, which would classify *X*′ as a “one-to-many” ortholog for both *X* and *U* [14].

At the same time, it is essential to highlight that the selection of a single paralog from two or more is justified given the primary objective of our method. Its primary goal is to identify genes whose emergence or disappearance during a specific evolutionary stage may be functionally linked to the appearance or disappearance of particular phenotypic traits at that stage. Since paralogs are distinct genes with similar but not identical functions, the "disappearance" of one of them in species *A*, as detected by our method, holds significance in achieving its goal. This event could potentially be associated with the emergence or disappearance of certain traits in species *R* compared to species *A*. For example, paralogs such as members of the HOX complex or genes of the CDX family, while producing proteins with similar molecular functions, exhibit different expression patterns, thus governing the development of distinct embryonic structures. Therefore, the fact that our method considers any gene from species *R*, including those with paralogs that meet the specified condition (*c*), as the sole ortholog of gene *X*′ in species *A*, should not be seen as confusing. Instead, it can be regarded as a convenient approach that enables the detection of changes in gene content that occurred during the stage of evolution associated with the disappearance or appearance of the phenotypic or physiological trait of interest.

### The genes identified by WINEGRET play critical roles in tail regeneration

In this work, we have shown that the *Xenopus laevis* orthologs of three tested genes selected from among 30 genes identified in our screening as lost in ancestors of reptiles and upregulated during tail regeneration in *Xenopus laevis* tadpoles are indeed essential for regeneration since, without their activities, regeneration is suppressed. In addition to demonstrating the validity of our screening, this finding is important in itself thanks to the following reasons.

First, C-C motif chemokine 4 and Eotaxin-like belong to the family of chemokine signaling factors that regulate the migration of a large number of cell types, in particular, cells involved in blastema formation during regeneration in cold-blooded and warm-blooded animals [11, 19, 34]. During the first and second days after tail tip amputation, several cell migration events take place, which could be associated with the enhancement of *c-c motif chemokine 4* and *eotaxin-like* expression observed in this early period of tail regeneration. Thus, shortly after amputation, there is intensive migration towards the wound surface of the innate immune system cells responsible for the initial inflammation and further clearing of damaged tissues [41]. Simultaneously, mesenchymal cells, notochord precursors, and myogenic cells begin to accumulate in regions near the amputation plane, adjacent to the edge of the amputated notochord, thus forming a bulk of cells for further proliferation and regeneration blastema formation [55]. Given all this, one may speculate that these chemokines regulate at least some of the above cell migration events preceding blastema formation.

Second, as a result of our study a novel, forth, family of Cu/Zn-superoxide dismutases called here SOD4 was established. As SOD4 proteins are extracellular, one may predict that they, like SOD3, catalyze the generation of H_2_O_2_ from O_2_^•−^, which is also produced extracellularly from molecular oxygen (O_2_) by NADPH oxidase (NOX) complex located on the cell membrane [3]. In turn, H_2_O_2_ is known to be signaling molecule that plays an important role in many intracellular and intercellular processes, particularly during regeneration of body appendages in anamniotes [5, 15, 40]. Interestingly, judging by Additional file 2: Table S2, the expression of the other three Cu/Zn superoxide dismutase genes, unlike *sod4*, is not increased in the regenerating tail tip. This indicates the unique role of *sod4* in regeneration, which is confirmed by the results of our experiments on *sod4* knockdown (Fig. 7E).

Obviously, the specific functions of the identified genes in regeneration should be subjects of more comprehensive investigation in future studies. At the same time, we can already argue that the loss of critical regulators of regeneration, such as c-c motif chemokine 4, eotaxin-like, and sod4 in reptiles’ ancestors further strengthens our hypothesis that the complete loss of some genes could be one of the reasons for the decrease in regenerative potencies in evolution [20–23, 30, 57].

## Conclusion

Evidence has accumulated that the loss or emergence of genes could underlie significant evolutionary transformations. The targeted search for such genes is a critically important but challenging problem. We propose a method called WINEGRET that allows one targeted screening for such genes. This method combines an original algorithm, allowing fast and wide-range screening for genes that were lost or emerged at any evolutionary stage, with testing the involvement of such genes in the development of a phenotypic trait of interest that was also lost or emerged at the same evolutionary stage. For the practical implementation of this method, we developed an effective computer program. To demonstrate the effectiveness of the developed method, we performed a proof-of-principle screening for genes that are absent in reptiles, birds, and mammals but present in fish and amphibians and participate in the regeneration of the amputated tail of *Xenopus tropicalis* tadpoles. As a result, 57 such genes were found; the expression of 30 of them significantly increased during regeneration while the expression of 27 significantly decreased. The critical roles in regeneration of three genes with increased expression, *c-c motif chemokine 4* (LOC100493779), *eotaxin-like* (LOC101733569), and a previously unknown protein (LOC100495179), were demonstrated in experiments on their morpholino-induced knockdown.

Regarding the third protein, we established that it belongs to the novel SOD4 family of Cu/Zn-superoxide dismutases, which we found in representatives of different types of Metazoa as well as in their nearest single-celled relatives. In sum, the results of this proof-of-principle study further strengthen our hypothesis that the loss of genes could be one of the reasons for the decrease in regenerative potencies in evolution.

## Methods

### Description of the three-steps algorithm for Task I

*Step 1. Weight of the gene homology* For each pair $\left( {x,y} \right)$ of proteins, the raw score $s\left( {x,y} \right)$ is calculated by BLAST using the BLOSUM62 matrix, where *x* acts as a query and *y* as a target. To do this for a large number of species, it is convenient to use a supercomputer. Then, we transform the raw score to the *normalized score* between the two proteins $w\left( {x,y} \right) = {{2s\left( {x,y} \right)} \mathord{\left/ {\vphantom {{2s\left( {x,y} \right)} {{\kern 1pt} \left[ {s\left( {x,x} \right) + s\left( {y,y} \right)} \right]}}} \right. \kern-0pt} {{\kern 1pt} \left[ {s\left( {x,x} \right) + s\left( {y,y} \right)} \right]}}$. Instead of taking the average to normalize the raw score, one can use either the smaller or larger of $s\left( {x,x} \right)$ and $s\left( {y,y} \right)$, which lead to largely equivalent results, not discussed here.

Next, we transform this score to the *weight* of the homology of genes *X* and *Y* using the formula $W\left( {X,Y} \right) = \max \left\{ {w\left( {x,y} \right)|x \in X,y \in Y} \right\}$, where $x \in X$ means that protein *x* is encoded by gene *X* in some variant of transcription or posttranscriptional processing. This method allows one to constrain this “protein–gene” correspondence with auxiliary conditions such as “consider only proteins *x* of a length close to that of the functional proteins coded by gene *X*” or “protein *x* must have important functional domains”. The magnitude of $W\left( {X,Y} \right)$ is normally between 0 and 1, but in relatively rare cases, it can exceed 1 due to the peculiarities of the protein alignment produced by BLAST. The greater the weight, the closer the homology of *X* and *Y*. Based on the $W\left( {X,Y} \right)$ values, we construct a matrix *W*, where each protein-coding gene *X* of a species is accompanied by no greater than *u* closest homologs *X*′ in each species *A*, including the same species. Our proof-of-principle study considers the species from Table 1 and uses *u* = 3, so the matrix *W* contains 55,834,366 gene pairs with nonzero weights, of which only 537 pairs (less than 0.001%)have weights greater than 1, with a maximum of 1.62.

*Step 2. Forward check* Let us consider the algorithm of this (and the next) step for our proof-of-principle example. For each gene *X* in the *Xenopus tropicalis* frog, we select *candidate genes* in species *A* that (*a*) are homologs *X*′ (for *X*) from the matrix *W* and (*b*) have a predefined number of so-called witness genes in the given-sized neighborhoods of *X* and *X*′. These witness genes in the two species also have to be homologs from the matrix *W*. We call the process of testing the conditions (*a*) and (*b*) a *forward check* of gene *X* (see Fig. 3A). Two witness pairs are provisionally shown in the Fig. 3A; the actual number of witnesses is an algorithm parameter. In our example, one witness is needed; i.e., the local synteny of genes *X* and *X*′ is postulated if at least one pair of homologous witnesses is found in the gene neighborhoods. A neighborhood size of 5 Mbp on either side of a gene was chosen. This can be related to the 3D structure of DNA, which includes topologically associated domains (TADs); the impact of enhancers is usually limited to the TAD size so that transcription control largely occurs within a TAD. Local synteny conservation is usually considered to be conservation within the same TAD. In mammals, the TAD size ranges from one to several Mbp [9, 10, 36, 49]. The program allows a taxon-specific neighborhood size.

The homology conditions for the candidate pair of genes (*X, X*′) and for witness pairs (*Y*, *Y*′), (*Z*, *Z*′)… may differ; let us call these the α*-* and β*-homologies*, respectively. The condition of the β-homology is applied to the witnesses and is usually stricter than that of the α-homology, which is applied to (*X, X*′). For instance, if the α-homology relies on a value of the parameter *u*, the β-homology can use a smaller *u* up to 1; i.e., the best hit (BH) or even bidirectional best hit (BBH) condition can be applied. In our example, for every species *A*, the α-homology uses *u* = 3, and the β-homology is the BBH condition.

In the example, Step 3 below is carried out for each species *A*, although the program allows it to be carried out only for selected groups or species. Assume that *X*′ is a homolog in species *A* of gene *X* in the reference species *R* (a frog in the example), and there exist the necessary number of witnesses near the candidate pair (*X, X*′) required for the forward check (Fig. 3A).

*Step 3. Backward check* This is a *backward check* of the pair (*X, X*′) from Step 2, where gene *X* is from *R* and *X*′ is from the current species *A*. The candidate *X*′ is *rejected* if the frog has another gene *U* such that it is α-homologous to *X*′ according to the matrix *W*, satisfies forward check conditions similar to those shown in Fig. 3A, and satisfies one more condition,

$$\left( c \right):W\left( {X^{\prime},U} \right) - W\left( {X,X^{\prime}} \right) > 0.$$

Condition (*c*) includes conditions (*a*) and (*b*) for the $\left( {X^{\prime},U} \right)$ pair. It can be rewritten in the form $W\left( {X^{\prime},U} \right) - W\left( {X,X^{\prime}} \right) > 0$ because we ensure the symmetry of the matrix *W*, as described in Methods. The meaning of condition (*c*) is that candidate *X*′ is rejected if it has an alternative homolog *U* in *R* that is closer than *X*; see Fig. 3B.

If the candidate *X*′ is rejected, the algorithm checks the next (in decreasing similarity order) candidate *X*′ for *X*. If all (of up to *u*) candidates *X*′ are rejected, then we say that the gene *X is absent* in *A* (does not have an ortholog); otherwise, it *is present* in *A* (has the ortholog *X*′). Notably, the genes *U* and *X* can be close or far in the sense of $W\left( {U,X} \right)$; see Additional file 8: Appendix 1.1. If the matrix *W* were nonsymmetric, then the gene *X*′ could be a more distant homolog of *U* than of *X* or even fail to be among the closest *u* homologs of *U*. In Methods, we describe an expansion of Step 3 that is also available in the program.

By testing the conditions (*a*), (*b*), and (*c*) for each protein-coding gene *X* in the reference species *R* against all lower and upper species as *A*, one may obtain a *list-1* of genes that are present in the lower species but absent in the upper species. These three conditions can also be tested with each middle species taken as *A*, which results in useful information regarding the presence or loss of the genes from list-1 in the middle species. Moreover, it is possible to enable the program to verify the *condition* that the gene *X* is not present in more than *k* species of the middle taxa, where *k* can be chosen specifically for each taxon. Importantly, by choosing $k = 0$ for all middle taxa, one may obtain a list of genes of the reference species *R* that are present only in species of the lower taxa and absent in all species of the middle and upper taxa.

Importantly, the program allows one to perform steps 2 and 3 of task I not only with a single species *A* but also with a whole group of species, *G*, using a group-specific combination of conditions and parameters, which is specified by the user in a convenient procedural language built into the program interface. Such a combination is formed with links “and”, “or”, and “not” between metaconditions of the form “gene *X* is present in the group of species *G*”. For different *G*, different parameters and additional conditions can be chosen. In general, the metacondition is satisfied for *G* if gene *X* is present in at least *k* species from* G* (*k* is a group-specific parameter).

In our proof-of-principle study, we screened all frog protein-coding genes against conditions (*a*), (*b*), and (*c*) to find those that are present in fish but lost in all placental mammals (Additional file 1: Table S1, Columns A–H). We considered gene *X* to be present in fish or in any other group of vertebrates shown in Fig. 1 if it has an ortholog in at least one species of the given group; however, the algorithm allows one to require the presence of a gene in any predefined number of species of the group. Notably, due to the high degeneracy of gene names in GenBank, Additional file 1: Table S1 contains many different genes under the same names (Column H). Moreover, some genes have names that are common for all vertebrates, such as *prolactin* and *Wnt11*. However, thorough manual verification of at least some of these genes using their accession numbers indicated in Additional file 1: Table S1, it was confirmed that none of these genes have orthologs in placental mammals.

For each frog gene in Additional file 1: Table S1, the number of fish species in which its orthologs were actually found is denoted by parameter *q* in Column L of Additional file 1: Table S1. In addition to *q*, we include in this table the parameters *m*, *n*, and *p* for the primitive mammals, birds, and reptiles, respectively (Columns I–K), to denote the numbers of species in these middle taxa in which the given gene was preserved. Since in our particular case, we performed the analysis for 3 primitive mammals (Columns M–O), 4 birds (Columns P–S), 6 reptiles (Columns T–Y), and 6 fish (Columns Z–AE) listed in Table 1, the values of *m*, *n*, *p*, and *q* do not exceed these bounds. Notably, the condition $m = n = p = 0$ easily provides one with a list of genes present only in anamniotes (fish and amphibians) and absent in amniotes (reptiles, birds, and mammals). In turn, the condition $m = n = 0$ yields the genes present only in cold-blooded vertebrates, i.e., in fish, amphibians, and reptiles, and absent in warm-blooded vertebrates, i.e., birds and mammals. Finally, the genes present in cold-blooded vertebrates and in birds but absent in mammals can be selected in Additional file 1: Table S1 with the condition $m = 0$.

All parameters of the algorithm that were used to compute our proof-of-principle example are specified in Methods, subsection **D**, and referred to as *standard parameters.* Notably, the rough estimate of the ortholog underprediction with these parameters is calculated as 1.1% on average. For the frog–human pair, our method correctly reports most of the orthologs provided in NCBI (except for 3.9%) and predicts orthologs for 12% of frog genes that are not provided there (see Additional file 8: Appendix 1.

Finally, it is important to note that our algorithm used in Task I is symmetric with regard to the selection of *R* and allows one to search for genes that either were lost or emerged in evolution. Indeed, one may choose an upper species, e.g., a mouse, as the reference species *R* and look for its “lost” genes in fish and amphibians. As a result, list-1 will be composed of genes acquired in evolution by upper species compared with lower species.

*Expanded Step 3* The right hand of the condition (*c*) equals 0. In general situation, this value is naturally considered as a parameter, $\lambda$, of our algorithm so the backward check condition is formulated as

$$\left( {c*} \right):W\left( {X^{\prime},U} \right) - W\left( {X,X^{\prime}} \right) > \lambda ,$$

where $\lambda \in \left[ {0,1} \right]$ is the algorithm parameter, which value can be empirically selected in each specific case. Recall that the condition (c*) includes the conditions (*a*)and (*b*)for the $\left( {X^{\prime},U} \right)$ pair. It can be rewritten in the form $W\left( {U,X^{\prime}} \right) - W\left( {X,X^{\prime}} \right) > \lambda$ due to the symmetry of our matrix *W*. As before, the meaning of condition (c*) is to reject candidate *X*′ if there exists its alternative homolog *U* in *R* that is closer than *X* at least by a certain *threshold*, determined by non-negative parameter $\lambda$; see Fig. 3B. The rationale to use the strict inequality in (c*) is to avoid possible cases when the homology weights of *X*′ versus *U* and *X* are equal or differ by a small value so that one cannot definitely say which gene, *X* or *U* is the actual ortholog of *X*′, or they are co-orthologs of it. As before, the test of condition (c*)is referred to as a backward check of the pair $\left( {X,X^{\prime}} \right)$ from Step 2, where gene *X* is from *R* and *X*′ is from the current species *A*. The candidate *X*′ is rejected if the frog has another gene *U* such that it is α-homologous to *X*′ according to the matrix *W*, satisfies the forward check condition similar to shown in Fig. 3A, and satisfies the condition (c*).

Then, by testing the conditions (*a*), (*b*), and (c*) for each protein-coding gene *X* in the reference species *R* against all lower and upper species being used as *A*, one can obtain a different list-1 of genes that are present in the lower species but absent in the upper species. Such list-1 differs from that obtained above for $\lambda = 0$ and depends on a value of $\lambda$ as well as subsequent lists obtained from the list 1. Namely, as $\lambda$ increases at fixed other standard parameters, these lists almost monotonously shorten; see Table 2, which demonstrates the dependence of the numbers of genes in list-1 to list-4 on a $\lambda$ value at standard other parameters.

**Table 2:** The lengths of list-1, list-3, and list-4 of frog genes found by the expanded algorithm depending on a value of $\lambda$ at other parameters being standard

$\lambda$	List-1	List-3	List-4
0.00	2392	268	109
0.01	2468	288	116
0.02	2484	276	107
0.03	2457	272	102
0.06	2374	269	97
0.09	2302	263	91
0.12	2237	255	83
0.15	2185	244	82
0.18	2114	226	78
0.21	2078	225	80
0.24	2024	221	77
0.27	2001	218	74
0.30	1977	216	74
0.33	1965	213	73
0.36	1949	210	70
0.39	1953	210	70
0.42	1928	205	68
0.45	1926	200	67
0.48	1923	201	67
0.51	1917	200	67
0.54	1921	199	66
0.57	1923	194	62
0.60	1927	194	61

Namely, list-1 consists of the genes that are present in fish and amphibians, and lost in placental mammals; list-3 contains only differentially expressed genes from the list-1; and list-4 contains only those genes from the list-3 that are present only in fish and amphibians and lost in reptilians, birds, and mammals from Table 1

**Fig. 8:**
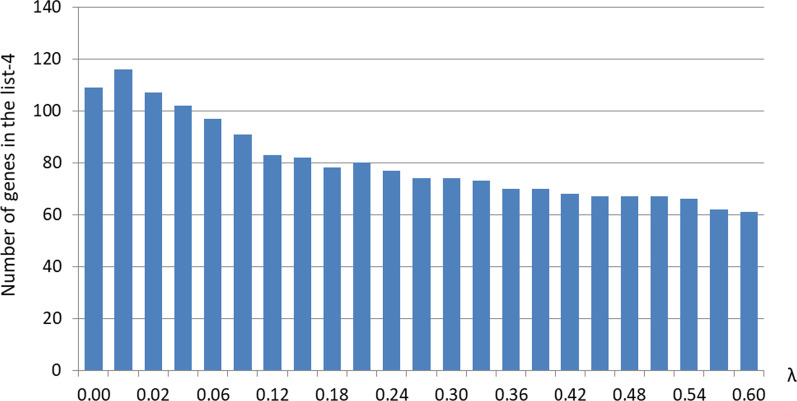
The length of the list-4 of genes depending on $\lambda$ value. For $\lambda$ from 0.02 to 0.42, the number of genes in the list decreases from 107 to 68. When $\lambda = 0$, this number is 109

For the species in our example, the comparison of the computation results led us to the range of 0.02–0.42 for $\lambda$ values. Indeed, at lesser or greater $\lambda$ the list-1 does not include genes which we considered as reliably lost, particularly from experimental data, or, vice versa, includes certainly preserved genes. Of course, the program allows one to compute the list-1 and other lists for any value of $\lambda$; results for $\lambda = 0$ were discussed above and provided in Additional file 1: Tables S1, Additional file 2: Tables S2, Additional file 3: Tables S3, Additional file 4: Tables S4, Additional file 5: Tables S5. However, for $\lambda$ values strictly less than 0.02, it is hard to say in some cases, which of two genes in the reference species *R*, *X* or *U*, is “substantially closer homolog” of *X*′ in species *A*. As a result, for a small $\lambda > 0$, list-1 includes, for example, the somatostatin-1 protein gene (LOC100486511), which in fact cannot be considered lost. On the other hand, for $\lambda = 0.45$, for example, the gene of fibronectin-like protein (LOC101733911), which, according to our manual verification, is indeed absent in reptiles, birds, and mammals and therefore seems to be a reasonable target for further experimental research, leaves the list-1. However, note that in case of any $\lambda$ the list-4 of genes that are present in fish and amphibians but absent in reptiles, birds, and mammals is quite short and does not exceeds 116 genes (for $\lambda = 0.01$)(see the plot of the number of genes in this list versus $\lambda$ in Fig. 8).

Notably, the advantage of pair $\left( {U,X^{\prime}} \right)$ over pair $\left( {X,X^{\prime}} \right)$ can be measured not only by the difference $W\left( {U,X^{\prime}} \right) - W\left( {X,X^{\prime}} \right)$ but also by the quotient ${{S\left( {U,X^{\prime}} \right)} \mathord{\left/ {\vphantom {{S\left( {U,X^{\prime}} \right)} {S\left( {X,X^{\prime}} \right)}}} \right. \kern-0pt} {S\left( {X,X^{\prime}} \right)}}$, where $S\left( {\, \cdot \,} \right)$ is the non-normalized raw score $s\left( {\, \cdot \,} \right)$ between proteins projected on respective genes as described above. In that case, condition (с*) looks like ${{S\left( {U,X^{\prime}} \right)} \mathord{\left/ {\vphantom {{S\left( {U,X^{\prime}} \right)} {S\left( {X,X^{\prime}} \right)}}} \right. \kern-0pt} {S\left( {X,X^{\prime}} \right)}} > \lambda_{1}$, where $\lambda_{1} \ge 1$ is other than $\lambda$ parameter of the algorithm. The program allows one to set either of these conditions. In the second case, similar lists of genes were obtained, which are not provided.

Recall that the final list of 57 genes produced by our algorithm at the standard parameters is provided in Additional file 5: Table S5.

### Search of lost frog’s genes

Using the set of 33 complete genomes listed in Table 1, we searched for amphibian genes that are retained in fish (also lower species) and lost in placental mammals (upper species). *Xenopus tropicalis* frog was chosen as the reference species *R*. The input data for Task II of the algorithm were obtained for the same species *R*. The list-1 of lost genes was initially based on only upper and lower species.

The proteomes of all species in Table 1 were coprocessed by a specially designed pipeline as follows (the pipeline is supercomputer-specific and is not provided):

1. For each proteome, a separate database was prepared from nonredundant proteins by *makeblastdb* with default parameters. From here on, we used the package BLAST + v.2.13.0 [4].
2. For each nonredundant protein *x* of each species *P*, we applied *blastp* to find all hits *y* in all species including *P*. Here, *x* was a query, and each proteome containing *y* was a target. The sparse matrix *V* was constructed with rows and columns corresponding to all proteins of all species. If a protein *y* is not a hit for the query *x*, such pair $\left( {x,y} \right)$ corresponds to an empty cell of the matrix. Otherwise, for each *x* that has a hit *y*, the cell $\left( {x,y} \right)$ of the matrix *V* contains the two characteristics, raw score $s\left( {x,y} \right)$ and *E*-value. Empty cells are implied to have these characteristics equal to zero and infinity, respectively. For this purpose, we run *blastp* with the parameters -outfmt “6 qseqid sseqid score evalue” -evalue 0.1 -max_hsps 1 -max_target_seqs 100 -use_sw_tback, other parameters were default.
3. The matrix *V* was symmetrized as follows: for nonempty cells $\left( {x,y} \right)$ and $\left( {y,x} \right)$, greater of the two values of raw score was substituted for lesser value, and lesser *E*-value was substituted for the greater one. If only one of the cells was empty, its characteristics were filled with values of another cell.
4. Normalize the raw score values $s\left( {x,y} \right)$ in *V* by transition to weights $w\left( {x,y} \right)$ as described in subsection “Description of the original method” of the Results section. Finally the matrix *V* is expanded with all existing copies of nonredundant proteins just duplicating the corresponding cells.
5. Transform the matrix *V* of proteins to the matrix *W* of genes *X* and *Y* as described in the mentioned subsection. Doing so, retain for each gene *X* no more than *u* genes of any species *A*: those genes *Y* that have the largest values of $W\left( {X,Y} \right)$. Extra nonempty cells to be deleted together with symmetric cells.
6. Convert the matrix *W* to the “table of homologs” format for the program *lossgainRZL*. For each gene *X* in a given species *P*, such table should not contain more than *u* the most similar (according to *W*) homologs *Y* in each species *A*; these homologs to be arranged by descending $W\left( {X,Y} \right)$ values.
7. The program provides the option of writing, in addition to the weight and E-value, the rank and back-rank of each homolog in the table of homologs in order to accelerate the subsequent search for lost genes. If one considers two homologous genes *X* and *X*′ in species *P* and *Q*, respectively, the *rank* is a serial number of *X*′ in the list of all homologs of *X* in *Q* provided that homologs are listed in descending weight order. The *back-rank* is a serial number of the gene *X* in a similar list of all homologs of *X*′ in *P* or (if the list lacks *X*) infinite value. Particularly, if *X*′ satisfies the BBH condition then both rank and back-rank equal 1.

The table of homologs thus constructed was used as input data for the program *lossgainRZL* [52].

### Analysis of RNA-seq data from GEO

We mapped the reads from SRA project SRP091865 (related with GEO project GSE88975) on the tenth assembly of *Xenopus tropicalis* genome, UCB_Xtro_10.0 (GCF_000004195.4), using the BWA-MEM aligner [35]. Then the alignments were sorted and indexed by samtools sort program. Differentially expressed (DE) genes were then identified by DESeq2 based on a table of the absolute (number of reads) and normalized (by transforming into the FPKM values) coverage of each gene. These tables were compared by the egdeR package [50]. Six groups of samples were compared: control (WT), 0 h post-amputation (hpa), 6 hpa, 15 hpa, 24 hpa, and 72 hpa. Genes for which the false discovery rate (FDR) level was below 0.01 were considered as significant and listed in Table S2. These genes were additionally checked against the general linear model [12] and found satisfying the same condition on FDR level (data are not provided).

### Standard parameters.

The following set of parameters called *standard parameters* was used in our proof-of-principle study:

- The reliability threshold of differential expression: FDR < 0.01 (less than 1% of errors);
- The number of *closest homologs* in species *A* with regard to the reference species *R*: 3 or less with the weight $W\left( {X,Y} \right) > 0.07$ and *E*-value < 1E-7;
- The size of neighborhood of the gene* X* or *X*′: 5 Mbp off either of genes (equal in all species);
- The sizes $r_{1} ,r_{2} , \ldots$ of neighborhood of the gene* X* or X* or X*′ in the three-species condition: 1, 2, 5 Mbp, respectively, off the gene (equal in all species);
- The number and quality of witnesses: at least 1 and BBH condition (equally for all species);
- For a frog gene *X* to enter in the list-1, it is sufficient to find the ortholog in at least one fish if such gene is absent, for our data, in placental mammals. If the gene *X* is present in a fish, usually it is present in several fish (see Additional file 1: Table S1, Column L).

Among these standard values, we varied the number of witnesses and neighborhood size; data are not provided since we did not observe significant distinctions, as opposed to the following generalization of Task I.

### Experiments with *c-c motif chemokine 4*, *eotaxin-like*, and *sod4* in regeneration of the amputated *Xenopus laevis* tadpole tail

#### Morpholinos injections

To test effects of *c-c motif chemokine 4* (GenBank: OCT81172.1), *eotaxin-like* (LOC108717158), and *sod4* (LOC121398191) downregulation, we injected directly into the fresh tail stumps of stage 42 tadpoles anti-sense vivo-MO, complementary to − 4 to  + 21 target site in mRNA of both *c-c motif chemokin4* homeologs (5′- CTCTTTTTAGGCTGGTAACCTCTTC), to -27–3 target site in mRNA of both *eotaxin-like* homeologs (5′- TTTCCCGAGACCTGAGTGATCCTAG), and to + 1– + 25 target site in mRNA of both *sod4* homeologs (5′- catcatttcggcaaggagcagcatt). Vivo-MO can penetrate through plasma membrane due to a unique covalently linked delivery moiety (Gene-Tools). Thus, local injection of vivo-MO at a certain developmental stage allows one to knock-down target gene at desired spatio-temporal parameters. After amputation, the anesthetized tadpoles were transferred from a 0.1 MMR solution with MS322 anesthetic to Petri dishes with a 3% agarose layer. For better spreading of vivo-MO, we injected them as 0.4 mM solution, in a mixture with the fluorescent tracer FLD, into the notochord and both fins in the direction of tail growth near the amputated edge. We repeated injections once per day during 1–4 dpa. After blastema formation, we injected solutions into the fins, notochord and the blastema. The *control-vivo-*MO (5′- GCAAGATTCCTCATTCAAAAGTCTC) was injected in the same way into the tail stumps of the control tadpoles. Statistical significance was calculated with the paired sample *t*-test and was set *P* < 0.05. Also, at 1–4 dpa, regenerated tails were collected for immunochemistry and total RNA extraction for qRT-PCR. To test the efficiency of *c-c motif chemokine 4*, *eotaxin-like*, and *sod4* vivo-MO, we utilized the approach, which we used for control efficiency of MO to other mRNAS in our previous papers [30, 31]). Namely, we co-injected it into the *Xenopus laevis* embryos with the synthetic mRNA encoding C-c motif chemokine 4, Eotaxin-like, and Sod4 tagged on C-end with myc-tag. To obtain these mRNA, we prepared plasmids containing *c-c motif chemokine 4*, *eotaxin-like*, and *sod4* cDNAs fragments, contained the MO target sites by PCR with the following pairs of primers:

*c-c motif chemokine 4*-myc-cloning dir 5′-TAAGAATTCGAGCAAGAGGAACTCAGAAAGA,

*c-c motif chemokine 4* myc-cloning rev 5′-ATACTCGAGGGCAACTTGATTGTTTGTTTTT;

*eotaxin-like*-myc-cloning dir 5′-TAAGAATTCAGTAAGTAGCCAGCTAGGATC,

*eotaxin-like-*myc-cloning rev 5′-ATATCTCGAGTGTCAGTACTGGAGCAGTTG;

*sod4*-myc-cloning dir 5′-GAGAGTCGGTTACAGAGGTAG,

*sod4-*myc-cloning rev 5′-ATATCTCGAGTTGGTTGACACGTGTATCGCAG;

The obtained three PCR fragments were sub-cloned using *Eco*RI and *Xho*I sites into pCS2-twsg1-myc plasmid (kindly provided by E. Parshina) instead of twsg1 coding region and checked by sequencing. In case of *sod4* cDNA fragment blunt cloning into EcoRI site was used. To obtain capped mRNAs, these plasmids were linearized by *Acc*65I and mRNAs were synthesized using mMESSAGEmMACHINE kit (Ambion).

For injection experiments, vivo-MOs were diluted to the final concentration of 0.3 mM; mRNAs were diluted to the final concentration of 25 ng/µL. The mRNA or MO solutions were mixed with FLD (Fluorescein Lysinated Dextran, 40 kDa, 5 mg/mL, Invitrogen) and 4–5 nL of the mixture were injected into single blastomeres at two-cell stage. Injected embryos were grown till stage 13, at which myc-tagged C-c motif chemokine 4, Eotaxin-like, and Sod4 in crude lysates of these embryos were analyzed by Western blotting as described in [23]. Coomassie stained gels were used as loading controls. As a result, we observed a strong decrease of the C-c motif chemokine 4, Eotaxin-like, and Sod4 bands in lysates of embryos co-injected with *mRNAs encoded myc-taged versions of all three these proteins* and vivo-MO to each of them compared to lysates of embryos co-injected with same mRNA and *control-vivo-*MO (not shown). This results confirmed the efficiency of the inhibition of *c-c motif chemokine 4*, *eotaxin-like*, and *sod4* mRNA translation by vivo-MO to these mRNAs.

#### QRT-PCR

QRT-PCR was performed and evaluated as we described previously in [20]. Briefly, for total RNA extraction from regenerating tail tips (1–4 dpa) and isolation we used respectively RNA extract reagent (Evrogen) and RNA isolation KIT (Evrogen). About 20–30 tails were used for each sample for total RNA extraction. The RNA quality and concentration were measured by Qubit^®^ fluorometer (Invitrogen). The reverse transcription (RT) of purified RNA samples was performed by M-MLV reverse transcriptase kit (Evrogen) according to the manufacturer’s guidelines. The qPCR was performed with qPCR-mix HS SYBR (Evrogen) was conducted on the DTprime 4 qPCR amplifiers (DNA-Technology) with a standard 40-cycle hot start program and the following pairs of primers:

*c-c motif chemokine4* dir: 5′-AAGGAGGACCTTCCCTGTGT and

*c-c motif chemokine4* rev: 5′-TTCCTTCATCTTCTGTCTAA;

*eotaxin-like* dir: 5′-CCAAAAGCCAAATGGGTGCT and

*eotaxin-like* rev: 5′-CCTTGATTGTTTTGGTTTCT;

*sod4* dir: 5′-GTGATACCATCATTCTGGTG and

*sod4* rev: 5′-ATGGTAGAATTCCATTCCTG.

The obtained PCR data were calculated by using the ΔΔCt method. The geometric mean of expression of ODC and EF-1alpha (housekeeping genes) was used for the normalization of genes expression levels as described in our previous works [22, 48]. The normalized PCR signal of the 0 dpa sample was taken as an arbitrary unit (a.u.) in each series. The data for each gene expression was calculated in 3–7 independent experiments.

### Supplementary Information

**Additional file 1: Table S1**. The list-1 of *Xenopus tropicalis* frog genes (2392 pcs) that are present in fish and absent from all placental mammals considered, at the standard parameters. For each frog gene, the characteristics *m* (number of primitive mammals that have this gene), *n* (number of birds that have this gene), *p* (number of reptiles that have this gene), and *q* (number of fish that have this gene) are specified. Columns: Protein (protein ID), Gene ID (frog gene ID), Chr, Start, End, +/- (gene coordinates), Symbol (gene symbol), Name (gene description), *m*, *n*, *p*, *q*, Monodelphis,…, Takifugu (19 columns with a gene ID in the respective species, if the gene is present in it, in the following order: primitive mammals, birds, reptilians, fish).

**Additional file 2: Table S2.** The list-2 of differentially expressed (DE) genes of *Xenopus tropicalis* frog (2007 pcs) identified by the RNA-seq analysis based on two models at FDR<0.01. Columns: Protein ID, Gene ID, Symbol (gene symbol), Name (gene description), Δt (two time points), logFC (Log-Fold-Change, logarithm of the gene expression ratio at two time points in “exact test” model), FDR (False Discovery Rate, probability of the type I error in “exact test” model).

**Additional file 3: Table S3.** The list-3 of *Xenopus tropicalis* frog genes (268 pcs) that are simultaneously: present in fish; absent in placental mammals; significantly change their expression level after the amputation of tail, at the standard parameters. For each gene, the characteristics *m*. *n*, *p*, *q* are shown, see details in the description of Additional file 4: Table S1. For the column descriptions ref. to Additional file 4 Table S1 and Additional file 5 Table S2.

**Additional file 4: Table S4.** The list-4 of *Xenopus tropicalis* frog genes (109 pcs.) that are simultaneously: present in fish; absent from reptilians, birds, and mammals; significantly change their expression level after the amputation of tail, at the standard parameters with *r*_1 _= 1 Mbp, *r*_2 _= 2 Mbp, *r*_3 _= 5 Mbp. For the column descriptions ref. to Table S1 and Table S2. In columns R, S, and T, the value 1 appears if the gene corresponding to this row meets the three-species condition for corresponding neighborhood size *r*_*i*_, otherwise the value is zero.

**Additional file 5: Table S5.** The final list of *Xenopus tropicalis* frog genes (57 pcs) that are simultaneously: present in fish; absent from reptilians, birds, and mammals; significantly change their expression level after the amputation of tail; meets the three-species condition for at least one size *r*_*i*_ of neighborhood, at the standard parameters with *r*_1 _= 1 Mbp, *r*_2 _= 2 Mbp, *r*_3 _= 5 Mbp. For the column descriptions ref. to dditional file 4 Table S1 and Additional file 5 Table S2.

**Additional file 6: Fig. S1.** Unrooted phylogenetic tree of SOD1, SOD2, SOD3, and SOD4 family proteins in the following jawless vertebrates as well as invertebrates: lamprey (*Petromyzon marinus*), lancelet (*Branchiostoma floridae*), sea star (*Patiria miniata*), fly (*Drosophila melanogaster*), priapulid (*Priapulus caudatus*), oyster (*Ostrea edulis*), hydra (*Hydra vulgaris*), trichoplax (*Trichoplax* sp. H2), sponge (*Amphimedon queenslandica*), choanoflagellate (*Salpingoeca rosetta*), filasteria (*Capsaspora owczarzaki*). Protein alignment and phylogenetic tree were built using MAFFT v7.511 [Katoh & Standley 2013] and IQ-TREE v2.2.0 [Minh et al. 2020, Kalyaanamoorthy et al. 2017] tools for proteins under the following accessions: XP_032800539.1 for Sod1Lamprey, XP_035686256.1 for Sod1Lancelet, XP_038059478.1 for Sod1Sea_star, NP_476735.1 for Sod1Fly, XP_014678703.1 for Sod1Priapulida, XP_048766647.1 for Sod1Oyster, NP_001274724.1 for Sod1Hydra, RDD37136.1 for Sod1Trichoplax, XP_003388880.1 for Sod1Sponge, XP_004342585.1 for Sod1Filasterea, XP_032828608.1 for Sod2Lamprey, XP_035687345.1 for Sod2Lancelet, XP_038059205.1 for Sod2Sea_star, NP_001286503.1 for Sod2Fly, XP_014666515.1 for Sod2Priapulida, XP_048766709.1 for Sod2Oyster, XP_002160626.2 for Sod2Hydra, RDD45336.1 for Sod2Trichoplax, XP_003389045.1 for Sod2Sponge, XP_004990675.1 for Sod2Choanoflagellate, XP_004365015.1 for Sod2Filasterea, XP_032822292.1 for Sod3Lamprey, XP_035676548.1 for Sod3Lancelet, XP_038068816.1 for Sod3Sea_star, NP_001036536.1 for Sod3Fly, XP_048756188.1 for Sod3Oyster, ABC25025.1 for Sod3Hydra, XP_032806339.1 for Sod4Lamprey, XP_035674789.1 for Sod4Lancelet, XP_038046810.1 for Sod4Sea_star, NP_733352.3 for Sod4Fly, XP_014672242.1 for Sod4Priapulida, XP_048746893.1 for Sod4Oyster, XP_047123939.1 for Sod4Hydra, RDD41664.1 for Sod4Trichoplax, XP_019863344.1 for Sod4Sponge, XP_004990415.1 for Sod4Choanoflagellate, KJE90024 for Sod4Filasterea.

**Additional file 7: Fig. S2.** Unrooted phylogenetic trees of SOD1, SOD2, SOD3, and SOD4 family proteins in the rectangular format with detailed branch bootstraps in the same species as in Fig. 5 and Additional file 1: Fig. S1.

**Additional file 8: Appendix 1.** A role of the backward check and similarity of genes X and U involved in it. Estimates of under- and overprediction rates of the proposed method.

## Data Availability

All data generated or analyzed during this study are included in this published article and its supplementary information files. The code of the software, the test example, and the documentation are available in the FigShare repository, https://doi.org/10.6084/m9.Figshare.20699158.
