# Supplementary figures and images for: Wide-scale identification of novel/eliminated genes responsible for evolutionary transformations
Source: Biol Direct. 2023 Aug 11;18:45. doi: 10.1186/s13062-023-00405-6 (PMC10416458; doi:10.1186/s13062-023-00405-6)

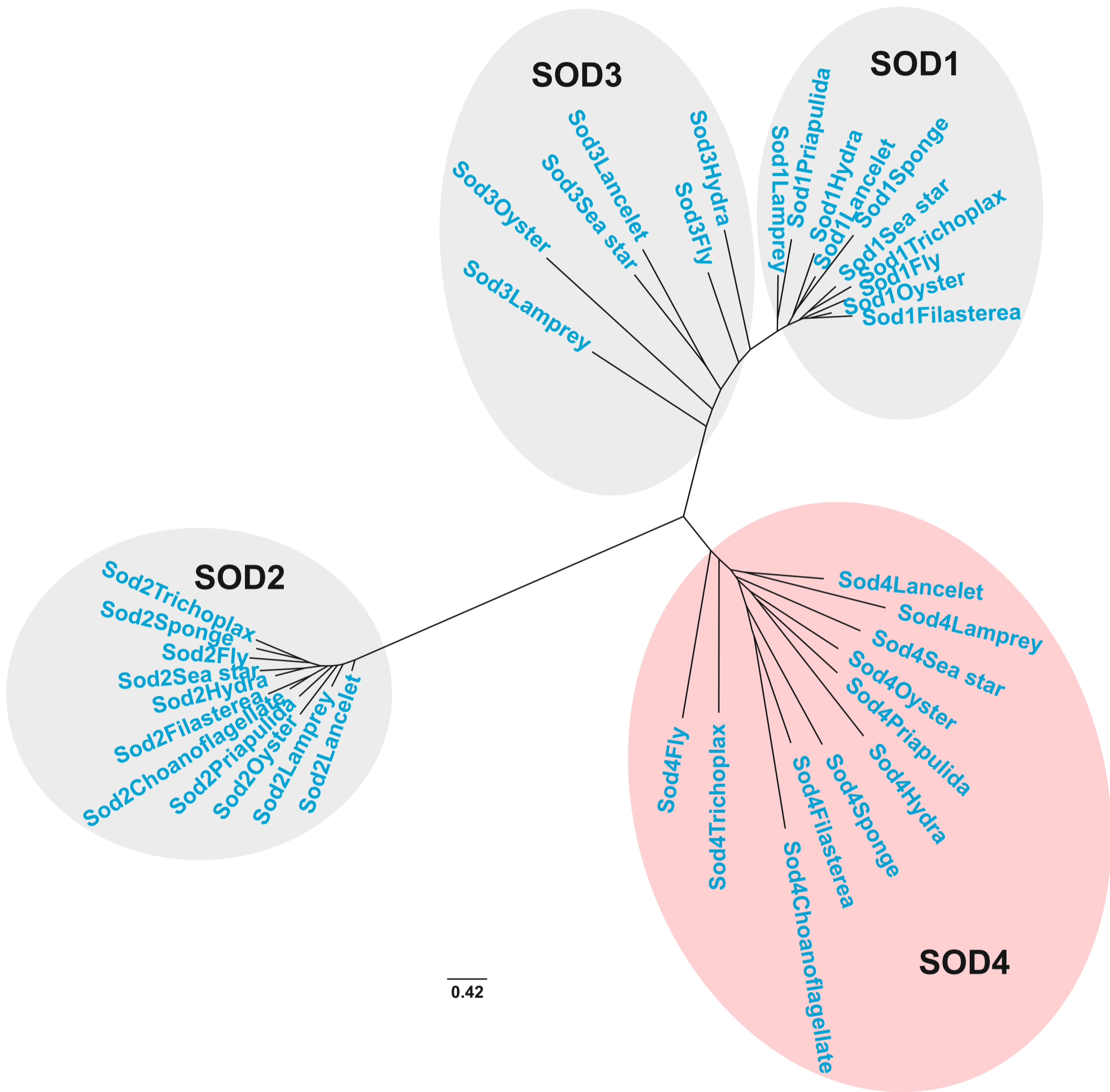

Supplement: Supplementary file 6 — Additional file 6: Fig. S1. Unrooted phylogenetic tree of SOD1, SOD2, SOD3, and SOD4 family proteins in the following jawless vertebrates as well as invertebrates: lamprey (Petromyzon marinus), lancelet (Branchiostoma floridae), sea star (Patiria miniata), fly (Drosophila melanogaster), priapulid (Priapulus caudatus), oyster (Ostrea edulis), hydra (Hydra vulgaris), trichoplax (Trichoplax sp. H2), sponge (Amphimedon queenslandica), choanoflagellate (Salpingoeca rosetta), filasteria (Capsaspora owczarzaki). Protein alignment and phylogenetic tree were built using MAFFT v7.511 [Katoh & Standley 2013] and IQ-TREE v2.2.0 [Minh et al. 2020, Kalyaanamoorthy et al. 2017] tools for proteins under the following accessions: XP_032800539.1 for Sod1Lamprey, XP_035686256.1 for Sod1Lancelet, XP_038059478.1 for Sod1Sea_star, NP_476735.1 for Sod1Fly, XP_014678703.1 for Sod1Priapulida, XP_048766647.1 for Sod1Oyster, NP_001274724.1 for Sod1Hydra, RDD37136.1 for Sod1Trichoplax, XP_003388880.1 for Sod1Sponge, XP_004342585.1 for Sod1Filasterea, XP_032828608.1 for Sod2Lamprey, XP_035687345.1 for Sod2Lancelet, XP_038059205.1 for Sod2Sea_star, NP_001286503.1 for Sod2Fly, XP_014666515.1 for Sod2Priapulida, XP_048766709.1 for Sod2Oyster, XP_002160626.2 for Sod2Hydra, RDD45336.1 for Sod2Trichoplax, XP_003389045.1 for Sod2Sponge, XP_004990675.1 for Sod2Choanoflagellate, XP_004365015.1 for Sod2Filasterea, XP_032822292.1 for Sod3Lamprey, XP_035676548.1 for Sod3Lancelet, XP_038068816.1 for Sod3Sea_star, NP_001036536.1 for Sod3Fly, XP_048756188.1 for Sod3Oyster, ABC25025.1 for Sod3Hydra, XP_032806339.1 for Sod4Lamprey, XP_035674789.1 for Sod4Lancelet, XP_038046810.1 for Sod4Sea_star, NP_733352.3 for Sod4Fly, XP_014672242.1 for Sod4Priapulida, XP_048746893.1 for Sod4Oyster, XP_047123939.1 for Sod4Hydra, RDD41664.1 for Sod4Trichoplax, XP_019863344.1 for Sod4Sponge, XP_004990415.1 for Sod4Choanoflagellate, KJE90024 for Sod4Filasterea. [file 13062_2023_405_MOESM6_ESM.pdf]

A

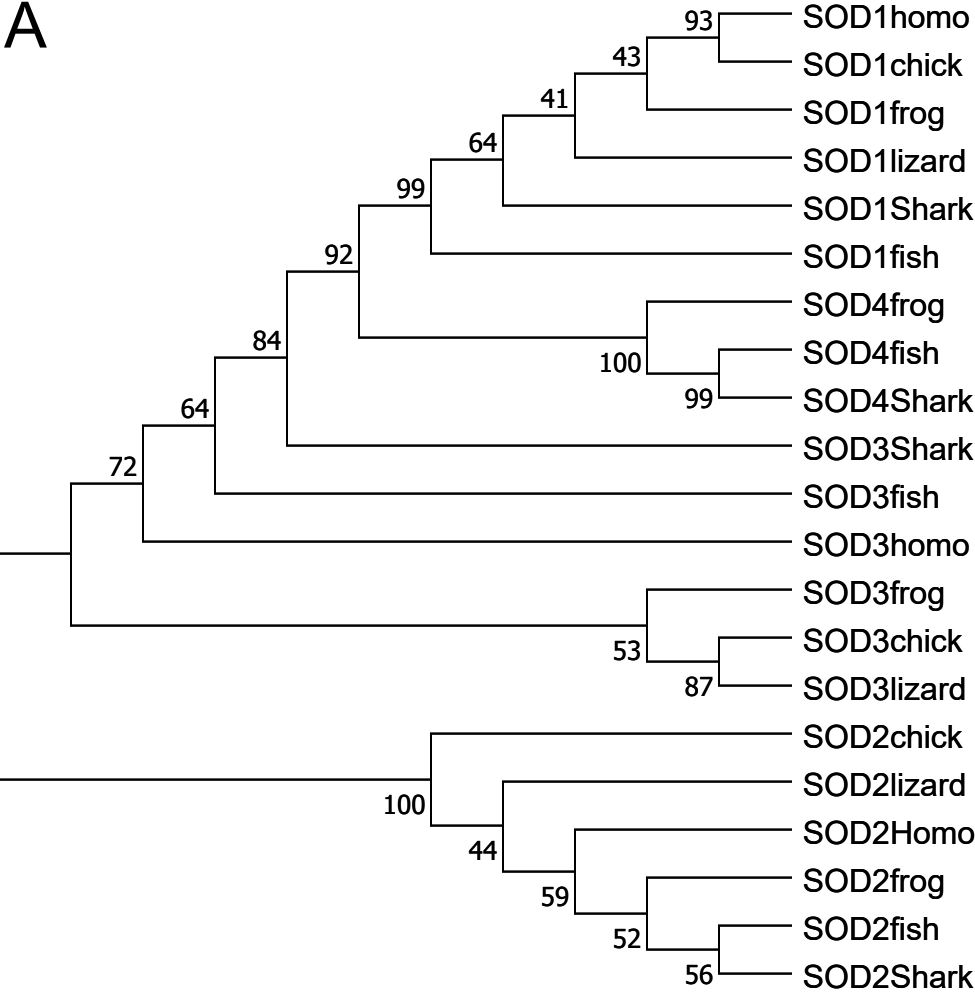

B

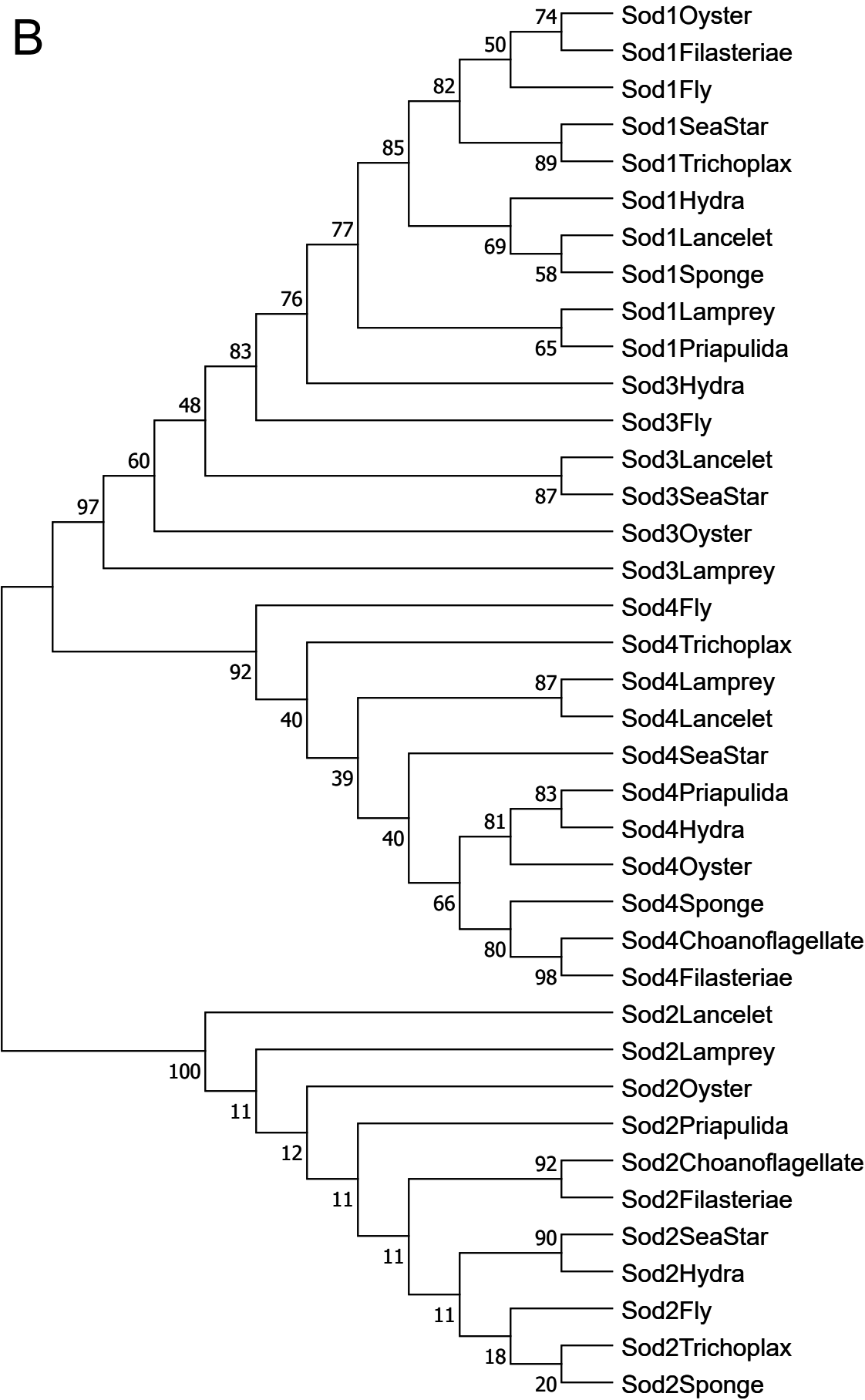

Supplement: Supplementary file 7 — Additional file 7: Fig. S2. Unrooted phylogenetic trees of SOD1, SOD2, SOD3, and SOD4 family proteins in the rectangular format with detailed branch bootstraps in the same species as in Fig. 5 and Additional file 1: Fig. S1. [file 13062_2023_405_MOESM7_ESM.pdf]
