# Supplementary material for: Wide-scale identification of novel/eliminated genes responsible for evolutionary transformations
Source: Biol Direct. 2023 Aug 11;18:45. doi: 10.1186/s13062-023-00405-6 (PMC10416458; doi:10.1186/s13062-023-00405-6)
Supplement: Supplementary file 8 — Additional file 8: Appendix 1. A role of the backward check and similarity of genes X and U involved in it. Estimates of under- and overprediction rates of the proposed method. [file 13062_2023_405_MOESM8_ESM.pdf]

Appendix 1

1. A role of the backward check and similarity of the genes *X* and *U* involved in it

Let us demonstrate a role of the backward check, i.e., condition (*c*) of the main text, by example of the human genome denoted *A* here, and genome of the reference species *R* that continues to be chosen tropical clawed frog *Xenopus tropicalis*. Using **standard** parameters of the algorithm **without a backward check** (ref. to main text), we found in *A* candidate homologs *X′* for 16747 of 21784 protein-coding genes *X* of frog (77% of genes). Thus, a candidate homolog *X′* of the given *X* should satisfy the conditions (*a*) and (*b*), one BBH witness is required within a radius of 5 Mbp, but the condition (*c*) is not applied. For the gene *X* we consider in *A* three best homologs or less if unavailable. For different *X* a different number of candidate homologs *X′* in *A* is found: one candidate for 13724 genes, two for 2130 genes, three for 893 genes, and for 5037 frog's genes there is no homolog **with a BBH witness** in human. Of all 14987 homologs *X′* found in human, only 12760 are homologs of exactly one frog's gene. Remaining 2227 *X′* are homologs of several frog's genes up to 71 pcs (Table 1). Finally, 4874 of 19861 protein-coding human's genes (25%) are not homologs of any frog's gene.

Table 1: The number of candidate genes *X′* in human that correspond to *n* different genes *X* in frog at the standard parameters of the algorithm. Here, the conditions (*a*) and (*b*) were met, but the condition (*c*) was not tested.

	n= 0	1	2	3	4	5	6	7	8	9	10–71
Number of genes *X′* with given number *n* of genes *X*	4874	12760	1457	290	117	91	65	39	37	22	109

The relation "genes *X* and *X′* make up a candidate homologous pair" for the pair of species "frog–human" is established for 20663 gene pairs. Among them, 11272 pairs have one-to-one relation, 3940 pairs – one-to-many, and 5451 pairs – many-to-many. One can see that the relation is ambiguous in 45% cases. In the case of differentially expressed (DE) genes with a FDR of less than 0.01, there is a one-to-one relationship between 992 gene pairs, one-to-many relationships for 562 pairs, and many-to-many relationships for 562 pairs too, among the total of 2116 homologous pairs. Thus, for DE frog's genes the number of candidate homologous pairs decreases almost tenfold, but the share of ambiguous pairs increases up to 53%.

Let us now consider the **standard** set of the algorithm parameters including the **backward check**. For each gene *X* in frog, homologs *X′* in human are searched by testing, besides the conditions (*a*) and (*b*), also the condition (*c*) or (*c**) with a parameter λ, i.e., both forward and backward checks are carried out. Results of the search at $\lambda = 0$, $\lambda = 0.02$, and $\lambda = 0.42$ are summarized in Table 2 with inclusion of similar data for just forward check. One can see that as the value of λ increases, the results tend to those of the backward check eliminated. In the upper half of Table 2 homologs were searched for all protein-coding genes in frog, and in the lower half – only for DE genes with FDR<0.01. One can see that the backward check significantly reduces ambiguity of the **ortholog**, i.e., in this study, a homolog that satisfies all three conditions (*a*), (*b*), and (*c*)/(*c**). The less the value of λ, the less the ambiguity of orthologs. This confirms the importance of the backward check (*c*)/(*c**).

Table 2: The results of the search of ortholog *X'* in human for protein-coding gene *X* in frog at standard parameters of the algorithm and depending on the value of λ .

	Description	Backward check: (*a*), (*b*), and (*c*) ($\lambda = 0$)	Backward check: (*a*), (*b*), and (*c**) at $\lambda = 0.02$	Backward check: (*a*), (*b*), and (*c**) at $\lambda = 0.42$	Without backward check: just conditions (*a*) and (*b*)
Among all protein-coding genes in frog	Number of frog's genes that have orthologs in human:	14439	14852	16593	16747
	• including those having just one ortholog	13856	14011	13847	13724
	• including those having two orthologs	464	645	1885	2130
	• including those having three orthologs	119	196	861	893
	• do not have ortholog in human	7345	6932	5191	5037
	Number of human genes that have orthologs in frog:	14820	14879	15009	14987
	• including those having just one ortholog	14760	14476	13112	12760
	• including those having two orthologs	32	259	1195	1457
	• including those having three orthologs	5	59	252	290
	• including those having four orthologs	5	34	103	117
	• including those having five or more orthologs	18	51	347	363
	• do not have ortholog in frog	5041	4982	4852	4874
	Maximum number of orthologs in frog for a human gene	45	46	71	71
	Total number of orthologous pairs between frog and human:	15141	15889	20200	20663
	• including one-to-one pairs	13634	13416	11756	11272
	• including one-to-many pairs	1348	1655	3447	3940
	• including many-to-many pairs	159	818	4997	5451
	The share of ambiguous pairs of orthologs	10%	16%	42%	45%
Among DE genes in frog	Number of frog's genes that have orthologs in human:	1251	1309	1541	1549
	• including those having just one ortholog	1177	1206	1142	1125
	• including those having two orthologs	63	78	260	281
	• including those having three orthologs	11	25	139	143
	Number of human genes that have orthologs in frog:	1332	1336	1371	1367
	• including those having just one ortholog	1329	1307	1215	1024
	• including those having two orthologs	3	25	107	200
	• including those having three orthologs	0	3	23	36
	• including those having four orthologs	0	1	8	28
	• including those having five or more orthologs	0	0	18	79
	Maximum number of orthologs in frog for a human gene	2	4	7	17
	Total number of orthologous pairs between frog and human:	1336	1437	2079	2116
	• including one-to-one pairs	1177	1169	1013	992
	• including one-to-many pairs	159	218	523	562
	• including many-to-many pairs	0	50	543	562
	The share of ambiguous pairs of orthologs	12%	19%	51%	53%

An ortholog in human is not always *superior* candidate homolog in respect of weight. Without a backward check or at $\lambda = 0.42$, 8% orthologs are not the best candidate homologs in this sense, and 10% orthologs if only DE frog's genes are considered. At $\lambda = 0.02$, these percentages are 5% and 6%, respectively, and at $\lambda = 0$ – 4% and 6%. For example, let us consider gene *arl5c* in frog (ADP ribosylation factor like GTPase 5C; NCBI gene_id: 733518; location 10:10455239..10464308;1). For this gene the best candidate homolog in human with the weight of 0.772 is the gene *ARL5B* (ADP ribosylation factor like GTPase 5B; gene_id: 221079; 10:18659335..18681639;1). The best but one homolog with the weight of 0.751 is *ARL5A* (ADP ribosylation factor like GTPase 5A; gene_id: 26225; 2:151798797..151828421;-1), and our method predicts the ortholog that has the third-highest weight of 0.721, gene *ARL5C* (ADP ribosylation factor like GTPase 5C; gene_id: 390790; 17:39156894..39166161;-1). The latter homolog is confirmed by a pair of BBH witnesses: *atp6v0a1* (ATPase, H transporting, lysosomal V0 subunit a1; gene_id: 100036694; 10:6264002..6315630;-1) in frog and *ATP6V0A1* (ATPase H transporting V0 subunit a1; gene_id: 535; 17:42458874..42522611;1) in human.

It might seem that in the course of backward check of gene X' as a candidate ortholog of gene X one should not only compare the degrees of similarity between the pairs $X – X'$ and $U – X'$, but also consider the similarity of the genes X and U themselves (see Fig. 4 in the main text). Our analysis shows that it is not true: irrespectively of the value of λ in the condition (c*), the candidate homolog X' should be rejected for X at both high and low similarities of X and alternative gene U in frog. Let us provide such examples in Table 3, where in each case the homology of genes is confirmed by the BBH witness pair within a neighborhood of 5 Mbp; the witnesses themselves are not provided.

Table 3: Examples of the candidate X' rejection for X at satisfied condition (c)/(c*) and different degrees of likeness between frog's genes X and U in this condition.

#	Role	Gene_id	Symbol	Name	Weight
1	X	548991	*tor1a*	torsin family 1, member A (torsin A)	$W(X, X') =$ 0.631
	U	733915	*tor1b*	torsin family 1, member B (torsin B)	$W(U, X') =$ 0.656
	X'	27348	*TOR1B*	torsin family 1 member B	$W(X, U) =$ 0.580
2	X	100486136	*pkd1l2*	polycystic kidney disease protein 1-like 2	$W(X, X') =$ 0.194
	U	101734692	*pkd1l3*	polycystic kidney disease protein 1-like 3	$W(U, X') =$ 0.219
	X'	342372	*PKD1L3*	polycystin 1 like 3, transient receptor potential channel interacting	$W(X, U) =$ 0.172
3	X	100496348	*dpysl5*	dihydropyrimidinase-related protein 5	$W(X, X') =$ 0.508
	U	100158561	*dpysl2*	dihydropyrimidinase like 2	$W(U, X') =$ 0.930
	X'	1808	*DPYSL2*	dihydropyrimidinase like 2	$W(X, U) =$ 0.508
4	X	496474	pdcd6ip	programmed cell death 6 interacting protein	$W(X, X') =$ 0.082
	U	100217320	ptpn23	protein tyrosine phosphatase, non-receptor type 23	$W(U, X') =$ 0.508
	X'	25930	PTPN23	protein tyrosine phosphatase non-receptor type 23	$W(X, U) =$ 0.076

For each of four examples in Table 3 the rejection of X' as an ortholog for X is biologically justified, because X' is an ortholog for rather U than X. In examples 1–3 genes X and U are two paralogs, and it is indeed U whose ortholog is X'. In example 4 X and U are even not paralogs, and X' is still ortholog for rather U than X. In examples 1–2 the condition (c) is met at a small $\lambda = 0.02$, and candidate X' is rejected at both high (0.580) and low (0.172) similarity weights between genes U and X. In examples 3–4 the condition (c) is met at a large $\lambda = 0.42$, and candidate X' is rejected at both high

(0.508) and low (0.076) similarity weights between *U* and *X*. Such a weight *W*(*X*, *U*) independence of the difference *W*(*U*, *X'*) – *W*(*X*, *X'*) results from a fact that *W*(*X*, *U*) much stronger correlates with a smaller weight *W*(*X*, *X'*) (correlation coefficient of 0.819) than with greater weight *W*(*U*, *X'*) (correlation coefficient of 0.05). These correlation coefficients were calculated on the totality of 145292 triplets (*X*, *X'*, *U*) observed between frog and human.

In each example our method finds other ortholog of gene *X* in human instead of rejected candidate *X'*; such ortholog has the same NCBI-annotation as gene *X* has. These orthologs are (numbers correspond to the examples in Table 3, and the weight of similarity between *X* and the ortholog is shown in parentheses: #1 gene_id:1861, *TOR1A*, torsin family 1 member A (0.657); #2 gene_id:114780, *PKD1L2*, polycystin 1 like 2 (gene/pseudogene) (0.464); #3 gene_id:56896, *DPYSL5*, dihydropyrimidinase like 5 (0.877); #4 gene_id:10015, *PDCD6IP*, programmed cell death 6 interacting protein (0.722). One can see that each of these genes is indeed the ortholog of the respective *X*.

2. Estimates of under- and overprediction of the proposed method

We choose tropical clawed frog *Xenopus tropicalis* as the reference species *R*, and besides it we consider 32 species from the main text denoted by *A* and listed in the first column of Table 4 starting from second line. Given gene *X* in *R*, we further denote *X'* its ortholog in *A*. Other columns of Table 4 contain the following data: genes – total number of protein-coding genes in species *A* that have at least two more genes at a distance less than 5 Mbp; *h* – the number of orthologs *X'* in *A* of the gene *X* in *R* that are found by our program among $u = 3$ closest homologs of *X* with **two BBH witnesses**, but *without a backward check*; *h'* – the same *with the backward check*; *s* – the number of orthologs *X'* counted in column *h* that have all witnesses located at one side of *X'*; *s'* – the same of *X'* counted in column *h'*. It should be noted that the standard parameters of our algorithm require at least one BBH witness per species considered, thus these orthologs are subject to stricter conditions, particularly, *X'* that have only one witness nearby is excluded. Finally, *rate* and *rate'* are squared ratios *s*/*h* and *s'*/*h'*, respectively. The primed denotations are those including the backward check.

Assuming that the ratio estimates the probability of *evolutionary ortholog* to lack a witness at one side of it within a given neighborhood, the squared ratio estimates the probability of the ortholog to have no witness in such neighborhood at all. Therefore, the probability of the evolutionary ortholog without witnesses can be estimated by the magnitude of *rate* (or *rate'* if the backward check is carried out). Such ortholog cannot be knowingly found by our method so the squared ratios are lower-bound estimates of its *underprediction* (or *type I error*) rate. Table 4 demonstrates that the average type I error rate equals 6.2% without the backward check, and 1.1% if the backward check is made, which argues in favor of its importance. Abnormally high rates of the type I errors are observed for the coelacanth and Tasmanian devil genomes, and, if the backward check is not carried out, for zebrafish genome as well.

Table 4: The lower-bound estimates of underprediction rate of the proposed method.

species	genes	h	h'	s	s'	rate	rate'
Xenopus tropicalis	21774						
Bos taurus	20871	21270	13560	4302	1010	0.041256	0.005548
Canis lupus familiaris	20099	21025	13454	4391	953	0.043708	0.005017
Equus caballus	20835	21662	13756	4137	890	0.036551	0.004186
Felis catus	20425	21318	13689	3984	806	0.035051	0.003467
Gorilla gorilla	20277	20813	13184	4420	1088	0.045248	0.006813
Heterocephalus glaber	19801	20201	13333	4611	1289	0.052235	0.009348
Homo sapiens	19859	21222	13683	4170	946	0.038683	0.004780
Loxodonta africana	20785	20210	13158	4337	1096	0.046262	0.006938
Macaca mulatta	20913	21167	13743	4014	955	0.036043	0.004829
Mesocricetus auratus	21276	21087	13516	4664	1070	0.049036	0.006267
Mus musculus	22179	21257	13556	4754	1181	0.050087	0.007590
Pan troglodytes	21238	20939	13604	3968	974	0.035990	0.005126
Sus scrofa	20462	21356	13581	4244	978	0.039614	0.005186
Monodelphis domestica	20492	20355	12943	4586	1143	0.050860	0.007800
Ornithorhynchus anatinus	18105	21719	13776	4187	862	0.037192	0.003915
Sarcophilus harrisii	19939	21228	13470	4470	900	0.044399	0.004464
Anas platyrhynchos	16735	20394	12914	3941	614	0.037405	0.002261
Gallus gallus	17933	20379	13073	3701	543	0.033076	0.001725
Meleagris gallopavo	16362	19844	11939	3974	543	0.040312	0.002069
Taeniopygia guttata	16414	19945	13235	3545	590	0.031712	0.001987
Anolis carolinensis	17611	19339	11918	5613	1746	0.084555	0.021463
Chrysemys picta bellii	19918	20673	13974	4129	1212	0.040023	0.007523
Crocodylus porosus	14702	18830	12384	3056	640	0.026839	0.002671
Notechis scutatus	16350	17751	12448	4516	1567	0.064884	0.015852
Pelodiscus sinensis	18382	18232	12305	5637	2359	0.095803	0.036753
Pogona vitticeps	18142	19617	13546	5349	2236	0.074707	0.027247
Callorhinchus milii	16750	21059	12173	5828	687	0.076910	0.003185
Danio rerio	25759	25549	10602	10428	1823	0.166892	0.029566
Latimeria chalumnae	18505	17721	11560	7144	3160	0.164090	0.074724
Oreochromis niloticus	29126	27602	11236	9541	1494	0.119717	0.017680
Oryzias latipes	22058	27502	10844	9807	1346	0.127408	0.015407
Takifugu rubripes	22045	29527	11299	9549	1134	0.104928	0.010073
**On average (except *X.trop.*)**	**19823**	**21275**	**12858**	**5156**	**1182**	**0.061609**	**0.011296**

The histogram of the type I error distribution is shown in Fig. 1. The vertical axis is the number of species, for which the magnitude of the type I error meets the inequality shown on the horizontal axis.

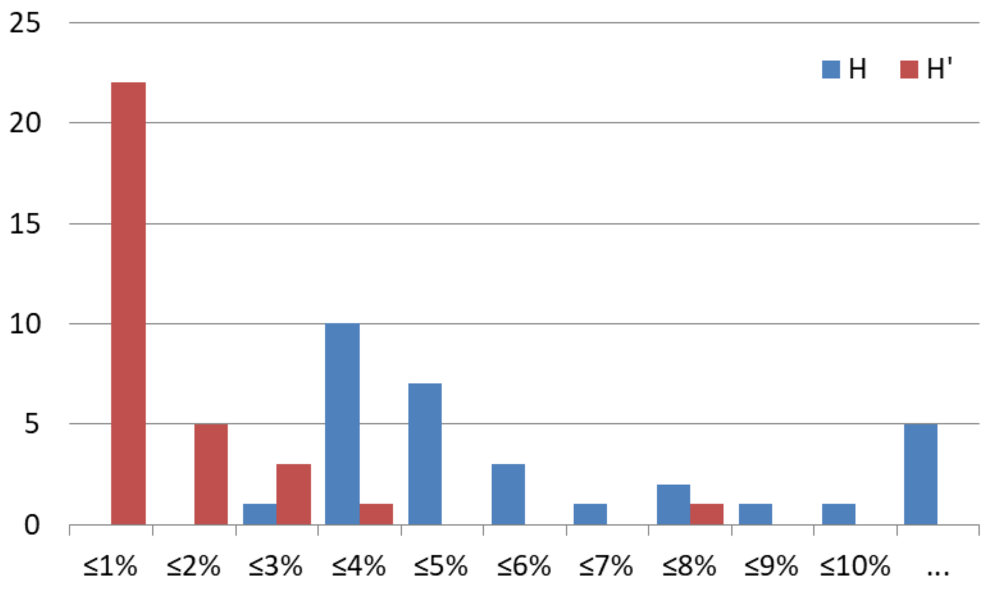

Figure 1: Distribution of the underprediction rate for 32 species from Table 4. Colors: blue means the type I error calculated without the backward check; red – the backward check is carried out.

The overprediction (i.e., the type II error) rate of the method may be estimated on the basis of direct comparison of the orthologs X' in human found by our method for frog's genes X with the table of orthologs available at ftp://ftp.ncbi.nlm.nih.gov/gene/DATA/gene_orthologs.gz. This table includes human orthologs for 13509 genes in frog. For almost all variants of parameters our algorithm detects in frog more orthologs of human genes than is present in the database (see column "XT genes" in Table 5). The comparison of the two sets of orthologs – predicted by our method and contained in the database – is provided in Table 5 depending on parameters of the algorithm. The table contains the following columns: # – a variant of the parameters; Homolog – what genes are selected as X': three best homologs (3-BH), best homolog (BH) or the best homolog in both directions (BBH); W_count – the number of witnesses; W_type – the selection of witness genes, same as chosen in Homolog or even stricter; T_back – whether the backward check is applied (at $\lambda = 0$) or not. Further, XT genes – the number of frog's genes, for which our method predicts an ortholog in human; =orth – the predicted ortholog coincides with that specified in the NCBI database; ≠orth – the prediction differs from ortholog in the database; N/A – no ortholog in human is present in the database. Based on these values, the error rates are calculated: Miss – NCBI-orthologs missing rate calculated as one minus the ratio of the number of orthologs coinciding with the database and the total number of NCBI-orthologs in the database; Wrong – the number of incorrectly (in respect of the database) predicted orthologs X' divided by the total number of NCBI-orthologs in the database; Type I – another estimate of the type I error rate, the sum Miss+Wrong; Type II – the type II error rate calculated as the number of orthologs X' found in human, but missing in the database, divided by the total number of orthologs X' found.

Table 5: Comparison of frog–human orthologs found by our method with NCBI-orthologs

	Selection detail (5 Mbp neighborhood)				Findings				Errors			
#	Homolog	W_count	W_type	T_back	XT genes	= orth	≠ orth	N/A	Miss	Wrong	Type I	Type II
1	3-BH	2	BBH	Yes	14104	12849	42	1213	4.9%	0.3%	5.2%	8.6%
2	3-BH	2	BBH	No	15979	12841	167	2971	4.9%	1.2%	6.2%	18.6%
3	3-BH	1	BBH	Yes	14440	13013	42	1385	3.7%	0.3%	4.0%	9.6%
4	3-BH	2	3-BH	Yes	13334	11908	147	1279	11.9%	1.1%	12.9%	9.6%
5	3-BH	2	3-BH	No	18283	11941	1231	5111	11.6%	9.1%	20.7%	28.0%
6	3-BH	1	3-BH	Yes	14383	12874	147	1362	4.7%	1.1%	5.8%	9.5%
7	BH	2	BBH	Yes	13770	12689	22	1059	6.1%	0.2%	6.2%	7.7%
8	BH	2	BBH	No	15187	12709	26	2452	5.9%	0.2%	6.1%	16.1%
9	BH	1	BBH	Yes	14019	12779	25	1215	5.4%	0.2%	5.6%	8.7%
10	BH	2	BH	Yes	13976	12682	25	1269	6.1%	0.2%	6.3%	9.1%
11	BH	2	BH	No	15958	12726	64	3168	5.8%	0.5%	6.3%	19.9%
12	BBH	2	BBH	No	13332	12510	20	802	7.4%	0.1%	7.5%	6.0%
13	BBH	1	BBH	No	13479	12609	23	847	6.7%	0.2%	6.8%	6.3%
14	BBH	0	BBH	No	14049	12919	61	1069	4.4%	0.5%	4.8%	7.6%

In terms of type I error rate, i.e., completeness and correctness of the NCBI-ortholog predictions, variant #3 of parameters achieves the best results, despite the smaller number of mispredicted orthologs if candidates are selected from BH or BBH homologs, because in the latter case more NCBI-orthologs are missed, leading to greater total type I error. One can see that backward check increases the prediction confidence at other conditions being equal.

An unsophisticated selection of BBH for candidate orthologs irrespective of witness existence (variant #14 of parameters) is inferior to #3 in both summands of the type I error rate. This rate is somewhat greater than the average rate estimated for human in Table 4. Our values of the type II error rate are apparently overestimated, since the mentioned database is not declared to be complete, and its completeness is essentially unknown. One might surmise that smallest type II error rates observed at homologs being selected only from BBH are explained by initial filling the database with BBH homologs and further curating it e.g. by addition a number of homologs which are not BBH. Noteworthy, it is **variant #3 of parameters in Table 5** that we used to obtain the list of lost genes provided in particularly Table S5. This is a set of parameters that we called **standard** in the main text.

Finally, let us verify these conclusions in the case of expanded backward check, i.e., when nonzero value of λ is applied in condition (c*), for example $\lambda = 0.02$ (Table 6). One can see that, where the backward check is applied, type I error rate decreases by 0.1%, while type II error rate increases by approximately 2%. Again, in this case variant #3 is the best in terms of type I error.

Table 6: Comparison of frog–human orthologs found by our method with NCBI-orthologs at $\lambda = 0.02$

	Selection detail (5 Mbp neighborhood)				Findings				Errors			
#	Homolog	W_count	W_type	T_back	XT genes	= orth	≠ orth	N/A	Miss	Wrong	Type I	Type II
1	3-BH	2	BBH	Yes	14459	12857	42	1560	4.8%	0.3%	5.1%	10.8%
2	3-BH	2	BBH	No	15979	12841	167	2971	4.9%	1.2%	6.2%	18.6%
3	3-BH	1	BBH	Yes	14853	13025	43	1785	3.6%	0.3%	3.9%	12.0%
4	3-BH	2	3-BH	Yes	13871	11951	177	1743	11.5%	1.3%	12.8%	12.6%
5	3-BH	2	3-BH	No	18283	11941	1231	5111	11.6%	9.1%	20.7%	28.0%
6	3-BH	1	3-BH	Yes	15012	12915	176	1921	4.4%	1.3%	5.7%	12.8%
7	BH	2	BBH	Yes	14018	12695	22	1301	6.0%	0.2%	6.2%	9.3%
8	BH	2	BBH	No	15187	12709	26	2452	5.9%	0.2%	6.1%	16.1%
9	BH	1	BBH	Yes	14315	12788	25	1502	5.3%	0.2%	5.5%	10.5%
10	BH	2	BH	Yes	14283	12696	26	1561	6.0%	0.2%	6.2%	10.9%
11	BH	2	BH	No	15958	12726	64	3168	5.8%	0.5%	6.3%	19.9%
12	BBH	2	BBH	No	13332	12510	20	802	7.4%	0.1%	7.5%	6.0%
13	BBH	1	BBH	No	13479	12609	23	847	6.7%	0.2%	6.8%	6.3%
14	BBH	0	BBH	No	14049	12919	61	1069	4.4%	0.5%	4.8%	7.6%
